# Advances in mosquito-borne disease surveillance using machine learning

**DOI:** 10.1016/j.nmni.2026.101757

**Published:** 2026-04-28

**Authors:** Mariana Geffroy, Juan Vicente Bogado Machuca, Gerardo Suzán, Fernando Esponda, Benjamin Roche

**Affiliations:** a Facultad de Medicina Veterinaria y Zootecnia, Universidad Nacional Autónoma de México (UNAM), Ciudad de México, Mexico; b International Joint Laboratory IRD/UNAM ELDORADO, Mérida, Yucatán, Mexico; c MIVEGEC, University Montpellier, CNRS, IRD, Montpellier, France; d National University of Caaguazú, Coronel Oviedo, Paraguay; e Departamento de Computación, Instituto Tecnológico Autónomo de México (ITAM), Ciudad de México, Mexico

## Abstract

Mosquito-borne diseases remain a major global health challenge, disproportionately impacting low- and middle-income countries. Despite traditional control and surveillance efforts, many of these diseases are resurging, driven by climate change, urbanisation, and global trade and travel. In recent years, machine learning, a subset of artificial intelligence, has emerged as a powerful tool for supporting the surveillance of MBDs. This systematic review, following PRISMA guidelines, examines 81 studies published between 2010 and 2024 to provide an overview of the current state of the art in applying machine learning techniques in the surveillance of malaria, dengue, Zika, chikungunya, yellow fever, and other mosquito-borne diseases. We highlight current trends in the use of machine learning techniques for forecasting, risk mapping, real-time disease monitoring, and vector/host ecology, and identify the most frequently used machine learning algorithms, including support vector machines, random forests, decision trees, and logistic regression. While machine learning models have shown promising predictive performance in some studies, their effectiveness depends on the availability, quality, and contextual relevance of the data. Gaps remain in model validation, implementation in low-resource settings, and inclusion of animal health data. Our systematic review outlines key findings, identifies research gaps, and proposes strategies for integrating machine learning in future mosquito-borne disease control efforts.

## Highlights

• Machine learning has several uses in mosquito-borne disease surveillance.
• Random forest and support vector machines are the most used techniques.
• Interdisciplinary data integration is essential for effective surveillance.

## 1 Introduction

Vector-borne diseases, especially those transmitted by mosquitoes, are a major public health challenge, with approximately 80% of the world's population at risk [1]. Mosquitoes of the *Aedes, Anopheles* and *Culex* genera can transmit at least 135 different pathogens affecting humans [2]. Still, the diseases of greatest concern due to their high prevalence and burden on regional health systems include malaria, dengue fever, chikungunya, Zika, yellow fever, West Nile fever and lymphatic filariasis. Malaria alone causes hundreds of millions of infections and at least a million deaths annually, while dengue, chikungunya, Zika, yellow fever, and lymphatic filariasis contribute to recurring outbreaks, long-term disability, and persistent public health challenges in endemic regions [[3], [4], [5], [6]].

Beyond their health consequences, these diseases have considerable economic and societal impacts, affecting the most disadvantaged populations and exacerbating existing inequalities, mainly affecting populations in tropical and subtropical regions [7]. Disease outbreaks disrupt health systems, burden economies through lost productivity and treatment costs and obstruct development. Annual costs associated with Aedes and their arboviruses range from US$3.29 to US$20.3 billion [8]; for malaria, the estimated costs are approximately US$4.3 billion [9,10]. These impacts highlight the urgent need for timely and effective strategies to anticipate and mitigate outbreaks.

This is where disease surveillance becomes essential: the ongoing, systematic collection, analysis, and interpretation of health data, followed by timely dissemination to the public [11]. It offers insights into disease behaviour based on location and season, identifying potential hotspots, and at-risk populations [12]. For mosquito-borne diseases, traditional surveillance methods typically focus on individual components, such as pathogen detection, vector monitoring, and case reporting [13,14]. Entomological surveillance involves monitoring mosquito populations through larval surveys, adult trapping, and taxonomic identification [15]. Additionally, epidemiological surveillance relies on case reporting and outbreak investigations. For certain mosquito-borne zoonotic viruses, surveillance can involve sentinel animals, which offer information about virus activity and increased risks to humans or other animals [16]. Entomological, epidemiological, and sentinel surveillance can inform outbreak prevention and detection, impede the spread of large-scale epidemics, assist policymakers and enable planning during outbreaks -such as insecticide deployment, vaccination campaigns, or other control measures to protect vulnerable communities- but are often labour-intensive, time-consuming, and sometimes complicated to apply.

Traditional surveillance and control methods, while essential, are no longer sufficient on their own. Multidisciplinary integrative approaches - such as ecological, evolutionary and socio-ecosystems frameworks-are needed to address complex health challenges. The One Health approach has been recognised as essential for strengthening the surveillance of MBDs, due to its emphasis on the interconnectedness of human, animal and environmental health, ultimately leading to more effective and sustainable solutions [17,18]. Many MBDs are zoonotic and circulate among multiple animal species, exhibiting diverse transmission cycles in which the roles of vectors, pathogens, parasites, and hosts can be examined [19].

In this context, One Health, or integrated surveillance, involves combining human epidemiological data, entomological vector monitoring, animal host or reservoir surveillance (including sentinel species), and environmental predictors such as land use, biodiversity, and climate [20]. Despite its conceptual appeal, operationalising One Health surveillance remains challenging, as data exchange hurdles and differing priorities between health professionals and institutions are among the main barriers to its implementation [21]. Field-collected data is highly valuable. Still, it is not always available, so there is a need for complementary tools and indirect methods that can process and combine heterogeneous data types - structured and unstructured datasets-, along with spatial, temporal, climatic, entomological, epidemiological and social variables, to generate accurate predictions.

The limitations of traditional mosquito control methods and disease surveillance, as well as those of integrated surveillance, open the door for emerging technologies such as Artificial Intelligence (AI), which may provide novel solutions or enhance existing approaches. AI refers to computational systems capable of mimicking human intelligence in tasks such as perception, learning, reasoning, problem-solving, and language understanding [22]. Its application has impacted various fields, and in the context of global health and infectious diseases, transforming disease control by supporting surveillance, policy development, diagnosis, and vaccine development [23,24].

One of AI's primary subfields, machine learning (ML), focuses on creating systems that can learn from data, discover patterns, and make data-based predictions and decisions. Standard ML techniques include classification, regression, and clustering using relatively simple models that can work with limited, structured datasets, allowing for a more straightforward interpretation and understanding of decision-making processes [25,26]. They also generally require less computational power and can be implemented with fewer data resources [27].

This review examines the prospects and challenges of employing ML methods in MBD surveillance, highlighting key advancements and areas for future research, and considers how an integrated perspective can enhance data integration across human, animal, and environmental domains to build more effective ML models.

## 2 Methodology

This systematic review was conducted in accordance with the Preferred Reporting Items for Systematic Reviews and Meta-Analyses (PRISMA) 2020 guidelines [28]. The protocol details the research questions, eligibility criteria, search methodology, study selection, data collection, and assessment procedures.

### 2.1 Research questions

This review aims to present the current state of the art in ML for the surveillance of MBDs. Thus, the research questions are the following:

Q1 What are the trends in using ML in the surveillance of MBDs?
Q2 Which ML techniques are used to surveil MBDs?
Q3 Are these novel methods for disease surveillance efficient and effective? What are the setbacks in their use?
Q4 What metrics are being used to evaluate the performance of these ML techniques?
Q5 What type of data is used to train these models? To which One Health domain does this data belong?

### 2.2 Eligibility criteria

To ensure the selection of relevant original studies, we included original research articles and conference proceedings published in English between 2010 and 2024 that evaluated the use of ML for the surveillance of MBDs. During the screening phase, studies were excluded if they were not related to MBDs (e.g. models for other vector-borne diseases or zoonotic diseases in which mosquitoes are not involved) or not focused on surveillance (e.g., studies about diagnosis or treatment). They were also removed if they did not meet the criteria for original research, such as opinion articles, letters to the editor, conference abstracts without complete data, or patents. We focused on ML approaches due to their increasing application in surveillance and their balance between predictive performance and interpretability. Eligible studies applied ML techniques and algorithms (e.g., support vector machines, random forests, decision trees). We decided not to include studies that use only deep learning (DL) architectures (e.g., Deep Neural Networks, Convolutional Neural Networks) to maintain model interpretability and comparability across studies. In public health forecasting, understanding the pathways linking predictors to outcomes is critical, yet DL models function as black boxes [29]. However, in some cases, we included studies employing DL only when such methods were directly compared to classical algorithms within the same study. Articles using only traditional statistical, mechanistic, or epidemiological models, without any machine learning algorithms, were also excluded. Studies that lacked sufficient methodological detail to assess the use of ML methods were also excluded.

### 2.3 Search methodology

The literature search was conducted using three databases: Web of Science (WOS), PubMed, and IEEE Xplore. Publications included those published from January 2010 to October 2024.

The search terms and phrases were consistent across all databases and tailored to each database's syntax, focusing on titles, abstracts, and keywords. All MBDs are included in the study, but only those considered by the WHO to be the most important [1] were included in the search. The search string was formatted as follows: (“mosquito-borne disease” OR dengue OR Zika OR malaria OR “West Nile virus” OR chikungunya OR “Japanese encephalitis” OR “yellow fever” OR “lymphatic filariasis”) AND (surveillance) AND (artificial intelligence OR machine learning).

### 2.4 Study selection

The initial searches returned 613 references. The deduplication and study selection processes were conducted using the Rayyan software [30]. Two independent reviewers screened titles and abstracts to identify relevant articles, applying predefined inclusion and exclusion criteria. The same reviewers independently assessed the full-text articles for inclusion in the study and extracted relevant information. At all stages, disagreements were resolved through discussion between the reviewers, and if a consensus could not be reached, a third reviewer was consulted. A PRISMA flow diagram detailing the identification, screening, eligibility, and inclusion of studies is presented in Fig. 1.

**Fig. 1:**
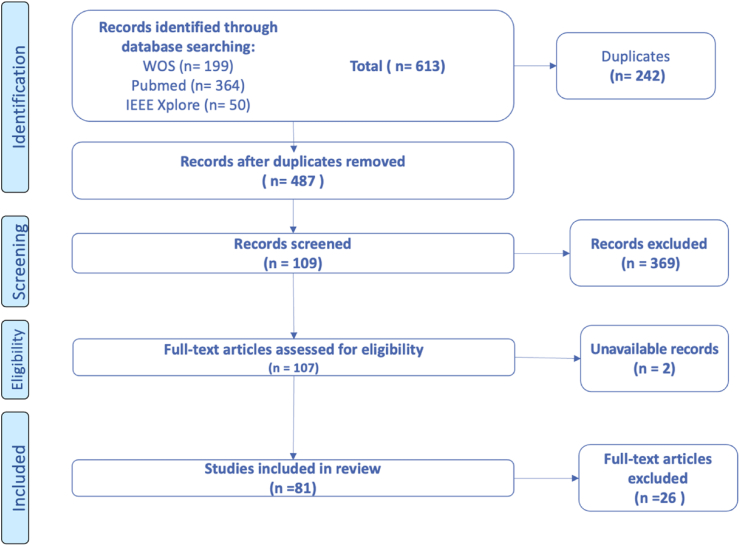
Flow chart illustrating the literature screening process following PRISMA guidelines.

### 2.5 Data collection

A data extraction spreadsheet was created using Google Sheets (Google, Mountain View, California, United States) to log relevant data from the selected articles, enabling simultaneous collaboration. The form included bibliographic information (study title, authors, year of publication), the pathogen studied, the country and region of study, dataset characteristics, date of data collection, type of predictors, One Health component considered, and the specific ML algorithms used. The effectiveness of these methods was also evaluated by extracting the evaluation methods employed in each study, noting the main outcomes and limitations identified.

### 2.6 Assessment procedures

Given the heterogeneity of study designs, data sources, and reporting practices across the included studies, a fully standardised risk-of-bias method was not feasible. However, to ensure methodological rigour, we conducted a structured risk-of-bias assessment inspired by the PROBAST framework (Prediction model Risk Of Bias Assessment Tool) [31], and we adapted it for surveillance ML models.

In this assessment we focused on three key domains relevant to ML models [1]: data quality, including sample size, sources and handling of missing data [2]; model development, including feature selection procedures, model specifications, transparency and risk of overfitting; and [3] model validation, including the type and use of validation strategies (e.g., cross-validation, hold-out, or external validation).

Each study was qualitatively assessed across these domains and classified as having low, moderate, or high risk of bias. This simplified approach allowed consistent evaluation of methodological limitations while accounting for the diversity of ML approaches included in this review. The overall score of the studies was determined by the highest risk identified in any single domain; for instance, a study with 'high risk' in Validation was classified as 'high risk'.

## 3 Results

### 3.1 Overview of the included studies

**Fig. 2:**
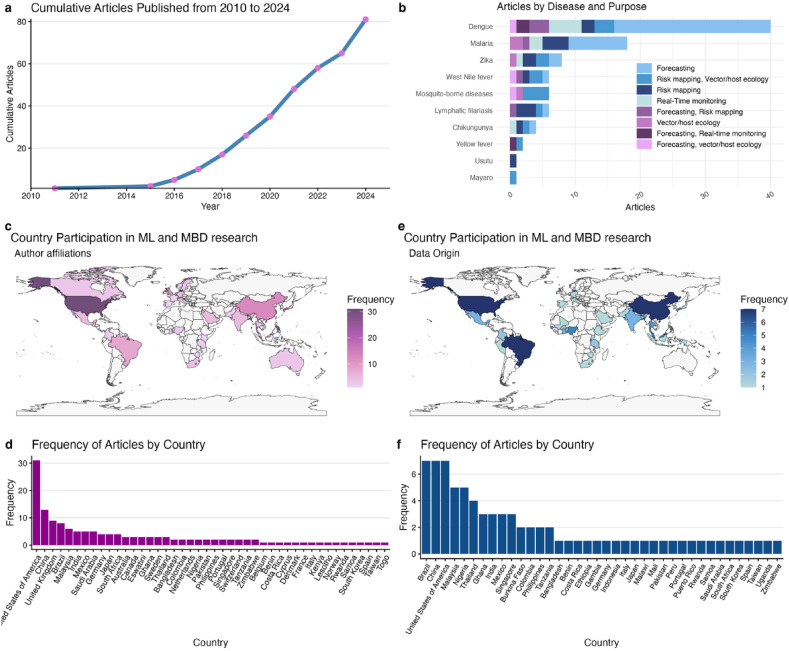
General overview of MBD articles using ML. (a) Cumulative number of published articles from 2010 to 2024, (b)Diseases researched in selected studies and their aim, (c-d) Countries participating in MBD surveillance research using ML, (e-f) Countries providing surveillance and MBD data to train ML models.

Eighty-one articles were selected in this review. Main characteristics are shown in Fig. 2 and Table 1. As shown in Fig. 2(a), growth remained minimal until 2016, then increased progressively in the following years, culminating in 81 cumulative publications by 2024.

**Table 1:** Overview of study characteristics, One Health data integration, predictor types and validation strategies in included studies (n = 81).

Study characteristics	Number of studies	%
Total number of studies	81	100%
**Country classification by income level (Data origin)**		
HIC	15	19%
MIC	49	60%
LIC	2	2%
Multi-setting (HIC + LMICS)	13	16%
No country reported	2	2%
**One health category used∗**		
Human data	71	88%
Animal data	25	31%
Environmental data	65	80%
Full One Health integration	15	19%
**Predictor types used for modelling∗**		
Epidemiological	68	84%
Climatic	62	77%
Geographic	40	49%
Demographic	37	46%
Entomological	24	30%
Socioeconomic	22	27%
Mobility	8	10%
Internet-based data	6	7%
**Model Validation**		
Cross-validation	43	53%
Temporal Cross-validation	13	16%
Hold-out validation	6	7%
Unclear/No validation	**19**	**23%**
External validation	**0**	**0%**

Study characteristics marked with a (∗) refer to those that had multiple categories used simultaneously.

We decided to create four broad groups—forecasting, real-time monitoring, risk mapping, and vector/host ecology—to encompass the diverse uses of ML in disease surveillance. Regarding the diseases studied in the articles, dengue dominated the ML research landscape, followed by malaria. In contrast, other MBDs, such as Yellow Fever, lymphatic filariasis, and Chikungunya, are markedly underrepresented. Japanese encephalitis was not identified, despite being included in the search parameters. In contrast, Mayaro and Usutu appeared in the search results, even though they were not explicitly searched for. In some cases, authors did not target specific pathogens, resulting in diseases being grouped under broader terms, such as “mosquito-borne diseases”, particularly when focusing on vector or environmental surveillance.

The analysis of global research contributions to the surveillance of MBDs using ML, was based on both author affiliations and dataset origins from the selected articles. For country counts of research contributions, countries based on author affiliation were counted once per article, regardless of the number of authors from the same country or affiliation. As illustrated in Fig. 2(c), the United States, China and the United Kingdom were the countries most frequently represented.

Furthermore, the origin of the datasets used in each model was analysed by identifying the countries that provided data for surveillance purposes. Only articles using original datasets were considered. In some cases, data were reported at a continental or global level. Due to difficulties in disaggregating these datasets into specific national sources, their contributions were not assigned to individual countries. Surveillance data features a strong representation from Brazil, North America, China, Malaysia, Thailand and several African nations (notably Nigeria and Ghana) (Fig. 2e). To enhance the analysis, countries were classified into income groups following the World Bank's classification [32]. Furthermore, most research was conducted in Middle-Income Countries (LMIC) (60%), followed by High-Income Countries (19%) and multi-setting studies (16%), which were those conducted in several countries with different classifications.

### 3.2 ML models, data types, metrics and validation used in MBD surveillance

Traditionally, ML is categorised into three main paradigms: supervised, unsupervised, and reinforcement learning [33]. Supervised learning involves training models on labelled data to perform predictive tasks. Unsupervised methods, in contrast, identify inherent structures in unlabeled data, whereas reinforcement learning involves an agent that learns an optimal policy through sequential interaction, guided by maximising a cumulative reward signal [34]. This review reports the use of 60 ML algorithms; most fall under the supervised learning category. Across the articles, 250 algorithms were applied, as most studies compared and evaluated multiple algorithms for the same task. This reflects a common practice in the field: comparing the performance of multiple algorithms on the same dataset. In contrast, a few relied on one or two models, often to address specific research objectives. A description of the various ML families, their algorithms, data requirements, and their uses is detailed in Table 2. Algorithms refer to the specific computational procedures or models used to analyse the data. Data requirements refer here to the minimum dataset characteristics needed for reliable model training and evaluation, including sufficient sample size, completeness of variables, and appropriate complexity for the modelling approach. Applications refer to the real-world or research contexts in which the algorithms are employed, including the type of problem addressed, domain of use, and expected outcomes.

**Table 2:** Machine learning algorithms found on reviewed records with their characteristics and uses.

Category/Family	Algorithms	Typical Use	Data requirements
Single Tree-Based Models	DT, CART, C5.0, J48, DTR, RT, LMT	Interpretable classification and regression methods	Low
Ensemble Methods	RF, RFR, QRF; GB, GBM, GBT, GBDT, GBR, GENBM; XGBoost, CatBoost, AdaBoost, BRT	Classification, regression, robust prediction	Moderate
Support Vector Machines & Kernel Methods	SVM, SVR, KRR, RBF tree	Classification and regression in high-dimensional spaces	Moderate
Linear & Regularized Regression Models	LR, MLR, LASSO, ElasticNet, GLR, glmnet, SDLR, PLSR, PLMR, PR, MARS, RNR	Regression, feature selection	Low-Moderate
Discriminant Analysis	LDA	Classification	Low
Clustering & Dimensionality Reduction (Unsupervised)	K-Medoids	Structure detection, feature reduction	Moderate
Instance-Based Methods	K-NN	Classification/regression using similarity/distance to neighbors	Low-Moderate
Naive Bayes & Probabilistic Models	BN, NB, NBIR, NBM	Fast probabilistic classification, often for text or categorical data	Low
Shallow Neural Networks	ANN, MLP, NN, BPNN; PNN	Simple pattern recognition, image analysis, time series	High
Deep Neural networks	CNN (including VGG16-19, ResNet50, GoogleNET, AlexNet)	Image/video, complex feature extraction	Very High
Specialized neural networks	ANFIS	Interpretable hybrids, imbalanced data	Moderate-High
Time series	ARIMA, SARIMA	Statistical forecasting	Moderate
Other/Specialized Methods	MaXEnt, JRIP, PLS-DA, GP, SRE, GAM	Varied: rule learning, time series, ecological niche modeling	Varies

*Abbreviations: AdaBoost = Adaptative boosting, ARIMA= Autoregressive integrated moving average, ANN = Artificial Neural Network, BN = Bayes Network, BRT = Boosted Regression tree, CART = Classification and regression tree, CatBoost = Categorie boosting, CNN = Convolutional neural networks, DT = Decision Tree, DTR= Decision Tree regression, ElasticNet = ElasticNet Regression, GAM = generalized additive models, GB = Gradient boosting, GBDT = Gradient boosting decision trees, GBM = Gradient boosting machine, GLR = generalized linear models, glmnet =Lasso and elastic-net regularized generalized linear models, GP= Gaussian processes, k-NN = K-Nearest neigbors, KRR = Kernel Ridge Regression, LASSO = Least absolute shrinkage and selection operator, LDA= Linear discriminant analysis, LMT= Logistic model tree, LR = Logistic Regression, MARS = Multivariate Adaptive Regression Splines, MaxEnt = Maximum Entropy, MLP = Multilayer perceptron, MLR = Multiple linear regression, NB = Naive Bayes, NBiR= Negative binomial regression, NBM = Negative binomial model, NN = Neural network, PLSR = Partial least squares regression, PLS-DA = Partial least squares discriminant analysis, PNN = Probabilistic neural network, PR = Polynomial regression, QRF = Quantile regression forest, RBF tree= radial basis function tree, RF = Random Forest, RFR = Random Forest regression, RNR = Ridge net regression, SDLR = Step-down linear regression model, SARIMA = Seasonal Auto-Regressive integrated moving average, SVM = Support Vector Machine, SVR = support vector regression, VGG = Visual geometry group, XGBoost= eXtreme Gradient Boosting machine* [34,35].

The top five most popular surveillance algorithms are detailed in Table 3. Random Forest (RF) was the most used algorithm, followed by SVM. The ensemble method, XGBoost, ranked third. The first two were also the algorithms that, when tested with the same datasets against others, performed the best.

**Table 3:** Top five ML Algorithms used in MBD surveillance, and the number of articles in which they were used.

Rank	Algorithm	Number of articles
1	Random Forest (RF)	38
2	Support Vector Machine (SVM)	23
3	XGBoost	14
4	Logistic Regression (LR)	13
5	Decision Tree (DT)	11

While identifying the most frequently used ML algorithms provides insight into current trends in MBD research, understanding how these models are evaluated is equally important. All methodologies must be assessed, and in ML, evaluation metrics determine a model's effectiveness in real-world applications. These metrics measure the model's ability to provide correct outcomes and indicate its performance while ensuring transparency and reproducibility. This approach also allows comparisons across different ML algorithms using the same datasets, thereby guiding the selection of the best model for a specific task. The metrics used in the evaluation of the models in selected articles are summarised in Table 4, along with their definitions, formulas, usage, and interpretation of their values.

**Table 4:** Evaluation metrics used in selected articles to measure model performance [36].

Metric	Definition	Formula	Best Used When	Interpretation
**Classification Metrics**				

Accuracy	Proportion of total correct predictions	$\frac{TP+TN}{TP+TN+FP+FN}$	Classes are balanced	Closer to 1 is better
Precision or PPV (Positive predictive value)	Of all predicted positives, how many are truly positive	$\frac{TP}{TP+FP}$	False positives are costly	Closer to 1 is better
Recall (Sensitivity)	Of all actual positives, how many were correctly identified	$\frac{TP}{TP+FN}$	False negatives are costly	Closer to 1 is better
Specificity	Of all actual negatives, how many were correctly identified	$\frac{TN}{TN+FP}$	Avoiding false positives	Closer to 1 is better
F1 Score	Harmonic mean of precision and recall	$2*\frac{Precision*Recall}{Precision+Recall}$	Precision and recall are both important. Good datasets with unbalanced classes.	Closer to 1 is better
AUC-ROC	Area under ROC curve: overall ability to distinguish between classes	Area under curve (graphical representation)	Evaluating discrimination across thresholds	0.5 no discrimination,0.7 ≥ is acceptable, ≥0.8 is good, ≥0.9 is excellent, 1.0 is a perfect classifier
Kappa (Cohen's kappa)	Agreement between predicted and observed classifications, adjusted for agreement occurring by chance	$\kappa =\frac{P_{o}-P_{e}}{1-P_{e}}$	Evaluating classification performance when class imbalance exists or when agreement by chance is a concern	Strength of agreement: <0 poor, 0.0 – 0.2 slight, 0.21-0.4 fair, 0.41-0.60 moderate, 0.61-0.80 good, 0.81-1.00 very good

**Regression metrics**				

R^2^ (R-Squared)	Proportion of variance explained by the model	$1-\frac{{SS}_{RES}}{{SS}_{TOT}}$	Evaluating model fit	Closer to 1 is better
RMSE	Root mean squared error; penalizes large errors	$\sqrt{\sum_{i=1}^{n}\frac{\left(\hat{y_{i}}-y_{i}\right)}{n}}$	Penalizing large prediction errors	Closer to 0 is better
MAE	Mean absolute error	$\frac{\sum_{i=1}^{n}\left|y_{i}-{\hat{y}}_{i}\right|}{n}$	Robust to outliers and easy to interpret	Closer to 0 is better
MAPE	Mean absolute percentage error	$\frac{100\%}{n}\;\sum_{i=1}^{n}\left|\frac{y_{i}-{\hat{y}}_{i}}{y_{i}}\right|$	Comparing percentage errors	Closer to 0% is better

Formula abbreviations: TP are the True Positives, TN are the True Negatives, FP are the False positives, FN refers to the False Negatives, SSRes refers to the sum of squares due to regression, SSTot is the total sum of squares, ŷ_i_ is the predicted value, y_i_ are observed values, n is the number of observations.

A critical component of machine learning reliability is model validation. Cross-validation was the most used validation approach (53%), followed by temporal cross-validation (16%) and hold-out validation (7%). Notably, 23% of studies reported an unclear validation strategy or no validation methods in their results. Of the 81 selected studies, none performed external validation (Table 1).

### 3.3 Risk of bias assessment

**Fig. 3:**
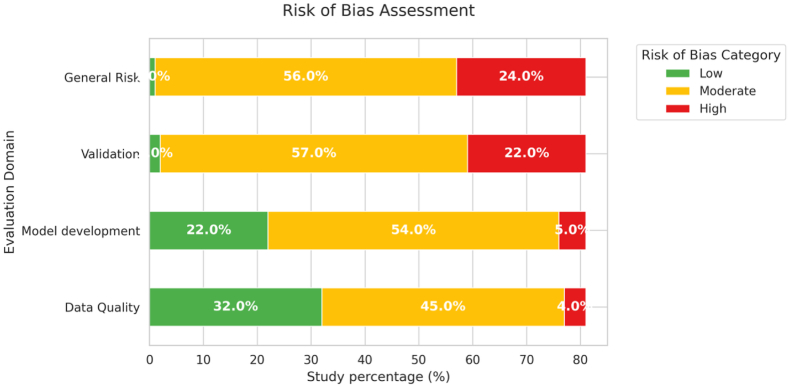
Summarised results of the risk of bias assessment for ML models used in MBD surveillance.

The risk-of-bias assessment revealed methodological limitations across the included studies (Fig. 3, Supplementary material 2). Overall, most studies were classified as having a moderate risk of bias, mainly due to limitations in model development and validation methods.

In the data quality domain, most studies were assessed as low to moderate risk, reflecting the use of well-defined data sources, although some studies used limited sample sizes or incomplete reporting. In contrast, model development was frequently associated with a moderate risk of bias, often due to insufficient information on feature selection and limited discussion of overfitting.

The highest risk was observed in the validation domain. Many studies relied on internal validation approaches such as cross-validation (53%) or hold-out methods (7%), while 23% of studies did not report any validation strategy. Notably, no studies performed external validation, highlighting a major limitation to the models' generalizability.

#### 3.3.1 Predictive variables used and their connection with the One Health concept

The main predictors used in the reviewed models were grouped into eight categories based on the nature and source of the data, and represent distinct dimensions of disease dynamics and data origin (Table 5).

**Table 5:** Predictor types with their definition and data examples found in selected articles.

Predictor type	Definition	Example
Climatic	Variables related to weather and meteorological conditions.	Temperature (max, min, mean), humidity, windspeed, precipitation, daytime/nighttime land surface temperature, sea surface temperature, soil moisture.
Demographic	Characteristics describing the structure and density of human populations.	Sex, age, urban/rural residence, population density, total population.
Entomological	Data related to vector abundance, distribution or infection rates.	Vector biodiversity, vector abundance, number of collections, number of traps, vector infection rates.
Epidemiological	Information on disease occurrence and dynamics.	Case numbers, incidence, prevalence, morbidity and mortality rates. Signs and symptoms.
Geographic	Spatial or environmental characteristics derived from maps or remote sensing.	Land use, normalised difference vegetation index (NDVI), enhanced vegetation index (EVI), ecosystem type, elevation, proximity to water bodies.
Internet-based	Data extracted from digital platforms such as search engines or social media.	Twitter (now X) data, Google Trends, search query data.
Mobility	Indicators of human or animal movement between areas.	Immigration and emigration data, land-surface travel times, number of domestic and overseas tourists, total passenger traffic.
Socio-economic	Variables describing social and economic conditions.	Per capita income, gross domestic product, household wealth, illiteracy, household size, housing type and condition, access to electricity, water, and sewage systems, etc.
Others	Variables that don't fit in the previously described predictor types.	Host or reservoir biodiversity and ecology, host-reservoir competence, knowledge, attitude and practices.

**Fig. 4:**
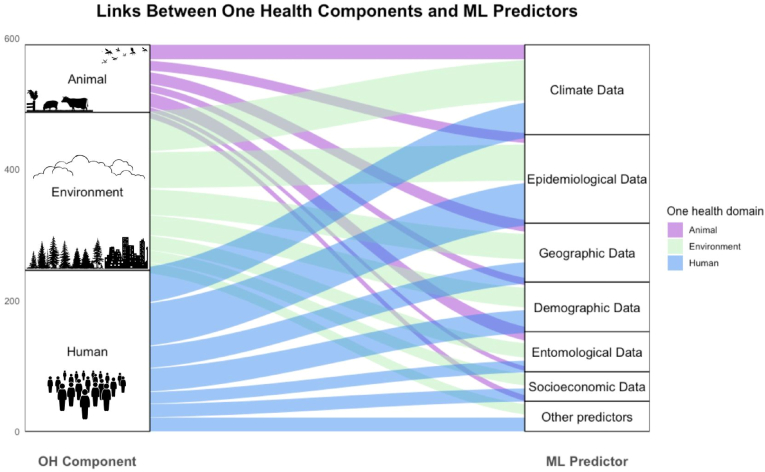
Relationship between the One Health domain and predictors used in ML models. On the left, we can find the One Health domains, and in the right column, the types of predictors used in the selected articles. The coloured bands represent the connection between these two. Other predictors that appeared fewer than 10 times across the selected articles are listed here: biodiversity data, human mobility data, internet search data, social media data, and clinical and diagnostic data.

This review examines how data for MBD surveillance models can be integrated across the human, animal, and environmental domains, in accordance with the One Health framework. The pattern is shown in Fig. 4, where the flow of predictors across One Health components shows a heavy reliance on human and environmental data—such as epidemiological, climatic, and demographic variables—while connections to the animal domain remain limited. This pattern persists even when we classify entomological data as part of the animal domain, treating vectors as animal arthropods that respond to ecological drivers.

As seen in Table 1 and Fig. 4, across most surveillance studies, there is a strong focus on human health data used in 88% of selected studies, particularly epidemiological information (84%) such as case incidence and mortality, as well as demographic (46%) and socioeconomic (27%) indicators to forecast disease. Environmental data were used to train 80% of the models with climatic variables (77%) such as rainfall, maximum and minimum temperatures, and humidity, along with geographic information on various land uses and ecosystems (49%). In contrast, the animal component of One Health remains notably underrepresented, with its use reported in only 31% of studies. Models encompassing all the components of One Health were identified in only in 15 studies (19%).

#### 3.3.2 Uses of machine learning in mosquito-borne disease surveillance

Different uses of ML algorithms for the surveillance of malaria, arboviruses, and lymphatic filariasis were identified in the 81 selected articles. Some of these models were employed to develop real-time surveillance systems, predict disease outbreaks and estimate case counts, identify key predictors of disease transmission, test remote sensing and social media data sources for disease forecasting, or enhance the accuracy of existing models by using other algorithms or ensemble models.

These groups are described in the subsections below, with examples, and a full summary of selected articles is available in Suppl 1 [105], [106], [107], [108], [109], [110], [111], [112], [113], [114], [115], [116], [117], [118], [119], [120], [121], [122], [123], [124], [125], [126], [127], [128], [129], [130], [131], [132], [133], [134], [135], [136], [137], [138], [139], [140], [141], [142], [143], [144], [145], [146], [147], [148], [149], [150], [151].

#### 3.3.3 Machine learning for forecasting mosquito-borne diseases

In public health, forecasting refers to predicting or estimating future trends, such as potential outbreaks or incidence rates, based on historical and environmental data [37]. The most popular models tested in selected articles were traditional ML algorithms, such as RF, XGBoost, SVM and LR, along with time-series models—such as ARIMA and SARIMA —. Malaria and dengue are the primary focus of most studies, which mainly apply climate and epidemiological case data to train the models.

Forecasting can be carried out at local, national, regional and global levels. For example, Benedum et al. [38] compared ML, regression, and time-series models to forecast weekly dengue case counts four weeks in advance and dengue outbreaks twelve weeks in advance in Iquitos, San Juan, and Singapore. Using climate data and weekly dengue case data, their results showed that ML models outperformed traditional methods, with RF achieving 21% and 33% lower error than regression and time-series models, respectively. Likewise, other variables, such as sea surface temperature, have been used as long-range predictors of malaria incidence in the province of Limpopo, South Africa [39]. ML classifiers trained on sea surface temperature patterns from the Indian and Pacific Oceans were able to forecast windows up to nine months ahead with 80% accuracy, beating the predictive power of local weather information.

At the national and subnational levels, in the USA, Keyel et al. [40] analysed 66 climate-related variables -mainly related to temperature, precipitation and soil moisture-alongside environmental and surveillance information using RF to identify the most important predictors. Their models significantly improved the prediction accuracy for WNV outbreaks, particularly when trained on hydrological data, such as streamflow and precipitation, which are critical for water-breeding vectors like *Culex*. In São Paulo, Baquero et al. [41] forecasted dengue cases using generalized additive models (GAMs) with meteorological variables and spatial-temporal smoothing functions. It accurately predicts large epidemics, with temperature, relative humidity, and accumulated precipitation being the key predictors. The model is suitable for real-world applications, as it can be updated and trained in just a few minutes.

Escalating to forecasting on a larger scale, Farooq et al. [42] used XGBoost to predict West Nile Virus (WNV) outbreaks in Europe and to explain their ecological drivers, identifying temperature and reduced water availability as critical predictors.

#### 3.3.4 Machine learning for the real-time monitoring of mosquito-borne diseases

The objective of real-time monitoring is to detect ongoing outbreaks or changes in disease prevalence using dynamic data streams and near-real-time collection, analysis, and reporting of surveillance data. This can be achieved in several ways, including social media and web-based analysis, sensor data, or automated surveillance systems. ML algorithms in this section are varied, encompassing LR, SVM, RF, LASSO, LDA, and XGBoost, while the main predictors were internet-based, entomological and epidemiological.

Social media allows people to share updates about their daily lives, including health-related topics. This information can be monitored, collected, analysed, and integrated into surveillance systems [43]. Google search queries about dengue symptoms and treatment could accurately predict dengue incidence in Singapore and Bangkok, surpassing traditional surveillance systems in both timing and accuracy [44]. LR performed well at predicting periods of incidence, and SVM worked better with higher incidences. Specific terms are used in searches, and these correlate with dengue incidence, as seen in China, where they forecasted the peak of an outbreak and allowed the tracking of dengue dynamics in several provinces [45]. Similarly, ML models applied to Twitter data (now X) enabled the early detection of seasonal outbreaks of dengue and flu using algorithms such as RF and SVM [46]. Nsoesie et al. [47] emphasised the value of Twitter for spatial and temporal surveillance, noting strong correlations between tweet volume and confirmed dengue cases across Brazilian states. Collectively, these studies show how digital and environmental data sources, combined with ML, can strengthen surveillance and response strategies for MBD. The effectiveness of social media for disease monitoring largely depends on socioeconomic factors, such as education, income, and differences in internet access between rural and urban areas; therefore, the information in some areas may not be sufficient to create accurate models.

These alerts can be used to develop decision-support tools and to communicate disease information to the public. Javaid et al. [48] developed a Web GIS–based dashboard that integrates real-time epidemiological data with vector mapping and environmental information in Pakistan. They achieved 93.7% disease accuracy using RF on 59,662 records with epidemiological, climatological, and socioeconomic data. Their platform enables district-level surveillance, geospatial case visualisation, and risk mapping for malaria, dengue and leishmaniasis. Similarly, Parikh et al. [49] created a web-based tool combining ML with visual analytics to detect the re-emergence of infectious diseases, including dengue and yellow fever. It was designed to maximise recall, at the expense of precision, especially for dengue, where a high number of false positives were produced. Despite these limitations, the tool analyses temporal trends and integrates multiple variables, giving users a view of outbreak dynamics while highlighting contributing causes.

#### 3.3.5 Machine learning on the risk mapping of mosquito-borne diseases

Risk mapping refers to spatial and ecological modelling and visualisation of disease occurrence and vector presence in certain geographic areas, assisting public health personnel and policymakers in identifying high-risk areas for targeted interventions [50]. Common methods include MaxEnt, RF, SVM, and ensemble models, often combined with geostatistics and Geographic Information Systems (GIS) to analyse distribution patterns. Diseases like malaria, dengue, and Zika are mainly investigated, along with lymphatic filariasis and emerging viruses such as Mayaro and Usutu.

Baak-Baak et al. [51] employed k-medoids clustering with mortality and morbidity data to categorise Mexican states by dengue risk levels and identify persistent dengue hotspots over a 13-year period, highlighting the need for local interventions in states with a high disease burden. Also in Mexico, Dong et al. [52] mapped the spatio-temporal dynamics of dengue, chikungunya, and Zika, revealing disease overlaps in high-incidence regions. They found that XGBoost performed best in terms of precision across all viruses, with socio-economic attributes having a greater impact on disease prevalence than climate attributes.

At a finer scale, Kabaria et al. [53] demonstrated how high-resolution satellite images and malaria surveys can be used to map intra-urban malaria risk. Malaria infection was heterogeneous across the city, with higher risks linked to proximity to dense vegetation, water, and swampy areas. Social and behavioural data can also enrich risk mapping efforts, where Rahman et al. [54], employed several ML models to map and predict *Aedes aegypti* abundance in 128 Thai households. They used entomological, socioeconomic, and landscape data, along with knowledge and practices related to dengue and climate change. When incorporating all these inputs, RF achieved the highest prediction accuracy, highlighting the importance of the surrounding environment and the adoption of preventative dengue practices.

In the context of lymphatic filariasis, Mayfield et al. [55] combined ML with geostatistics to identify residual infection clusters in Samoa, significantly improving surveillance and sampling efficiency. The odds ratio for identifying an infected individual in a household at a predicted high-risk compared to a predicted low-risk location was 10.2 (95% CI 4.2–22.8). In Nigeria, a quantile regression forest model was employed to predict the distribution and prevalence of lymphatic filariasis using antigenemia and microfilariaemia data, along with a range of continuous gridded environmental and climate data. The resulting maps predicted a heterogeneous distribution, estimating the mean number of infected individuals to be 8.7 million for antigenemia and 3.3 million for microfilaria prevalence [56].

Less common arboviruses have also been modelled. Lorenz et al. [57] applied MaxEnt modelling to forecast potential Mayaro virus transmission zones in South America, identifying biome type, rainfall, and elevation as the main risk drivers, while observing a geographic shift and the virus becoming more “urbanized”. Finally, Jiang et al. [58] used BPNN, GBM and RF models and identified four high-risk regions for Zika transmission: Southeastern North America, Eastern South America, Central Africa, and Eastern Asia. The BPNN model demonstrated higher predictive accuracy and lower uncertainty.

Risk mapping can also be applied in non-endemic settings, such as predicting dengue importation into Europe using ML and air traffic data using XGBoost [59]. In this study, the most important variables were the source country's incidence rate, population size, and air passenger volume. A similar approach was used in China, modelling malaria importation risk with XGBoost and RF models, using the records of imported malaria cases and socio-economic and connection features of malaria origin countries [60]. This highlights the usefulness of these models for border surveillance and control.

#### 3.3.6 Machine learning on vector/host ecology of mosquito-borne diseases

Vector and host ecology studies try to understand the biological and ecological dynamics of mosquito vectors, pathogens, and their hosts, which is essential for predicting disease emergence and transmission [61]. Several studies have applied ML and ecological modelling to define vector habitats, species distributions, and transmission cycles. The main ML algorithms used were XBoost and RF. The aims of some studies on vector and host ecology also overlap with risk mapping and forecasting.

Candeloro et al. [62] developed an XGBoost model, incorporating environmental data, animal case information from bird and horse hosts, and vector data to predict WNV circulation in Italy. Their model forecasted the spatio-temporal spread of WNV virus two weeks in advance with 84% accuracy, classifying provinces by infection status. Similarly, Beeman et al. [63] used ensemble ecological niche models to estimate the probability of West Nile virus (WNV) occurrence in Florida, utilising remote sensing and sentinel chicken surveillance data. The ensemble model demonstrated excellent predictive capability, finding that sentinel chicken coop locations enabled accurate identification of WNV exposure sites. Judson et al. [64] combined ecological niche modelling with historical outbreak data to investigate yellow fever transmission in Ghana, differentiating between sylvatic, savanna, and urban cycles based on ecological and seasonal patterns. Vegetation, land cover, climate factors and the presence of non-human primates were used as inputs to train the model, as they are known risk factors for yellow fever.

Other studies focused more directly on vector distribution and ecology. Alexander et al. [65] examined microgeographic determinants of Ae. Aegypti abundance in Miami, using RF, reveals that land use and urban socio-demographic features influence mosquito dynamics differently in neighbourhoods affected by Zika. Mosquitoes in different microgeographies show varied responses to meteorological factors. In Nigeria, Eneanya et al. [66] applied GBM and RF models to map environmental suitability for lymphatic filariasis and estimate populations living at risk. They identified spatial heterogeneity in vector habitat suitability driven by topography, climate, and vegetation and 110 million individuals at risk.

Focusing on global vector distributions, Ding et al. [67] employed SVM, RF, and GBMs to model the worldwide occurrence of *Aedes aegypti* and *Aedes albopictus*. RF was the best-performing model, revealing significant geographic and climatic factors that support their co-occurrence and expansion of their global range. Vector distribution and expansion can also be forecasted with ML. For instance, Georgiades et al. [68] used climate and environmental variables, global vector surveillance data, and an ensemble of XGBoost and RF classifiers to project future habitat suitability for *Aedes albopictus*. They identified a significant expansion towards the poles by 2100, and an increase in the number of months suitable for *Ae. albopictus,* especially in high-emission climate scenarios.

## 4 Discussion

The application of ML to MBD surveillance has steadily increased over the past decade. The use of ML for surveillance can aid in predicting diseases in advance, support real-time disease monitoring across various data streams, and provide information to identify and delineate areas and populations at higher risk of contracting MBDs. All these functions support decision-making, resource allocation and contribute significantly to improving health in at-risk populations.

Studies on ML and dengue surveillance dominate the research landscape, closely followed by those on malaria, highlighting their global burden and relevance to public health [1,6]. In contrast, research on other emerging MBDs remains limited, which could be linked to reduced funding, lower disease incidence in some regions that affect research priorities, lack of historical data, or less established surveillance systems [69]. At the same time, surveillance efforts rely on long-term field monitoring programmes, entomological data, or national public health reporting systems-resources that are generally more developed for malaria and dengue than for other MBDs [[70], [71], [72]]. However, they are not perfect due to certain limitations, like a high proportion of asymptomatic or unreported infections accompanied by coinfections and complex serotype or virus interactions [73], which create multiple pathways of transmission in which obtaining disease-specific data to train ML models might be challenging, especially in the case of emerging viruses.

Both high-income and low- and medium-income countries have been actively participating in the research on these technologies, either by providing information, samples, or methodologies to address these issues. This geographic distribution may reflect the tendency for research projects to be primarily funded by high-income countries, with the United States alone reportedly supporting approximately 37% of global malaria research and the United Kingdom 8% [6]. Similar trends are observed in arbovirus research [74]. The distribution could also be attributed to the significant investment in AI research in countries such as the United States, China, and the United Kingdom, which are recognised as AI powerhouses, leading global AI use, investment, infrastructure, and talent [75]. The contributions of countries such as Brazil, Malaysia, and Nigeria, among others, can be attributed not only to growing national investments in science and technology but also to the high local incidence and burden of MBDs [76,77], which shape research priorities and drive international collaboration.

Regarding the algorithms used, we observed a wide range that can be integrated into the MBD surveillance toolkit. It is important to note that the best-performing ML classifier will differ across datasets. Among the reviewed studies, RF was the most frequently used algorithm, likely due to its ability to handle complex, high-dimensional data and produce reliable results even with limited or imbalanced datasets [78,79]. It was followed by algorithms like SVM, which excelled in classification tasks, and XGBoost, which was well-suited for managing spatiotemporal and multivariate environmental datasets and consistently ranked among the top performers.

We also acknowledge that many of the reviewed ML models report good performance, but the evaluation approaches and metrics used were highly heterogeneous across studies and not applied consistently. For the evaluation of these models, the use of specificity and area under the ROC curve (AUC-ROC) was emphasised to minimise false outbreak alarms and improve geographic or temporal prediction accuracy. Regression tasks, such as predicting mosquito abundance or disease incidence, often use metrics like RMSE or R^2^ to assess whether model predictions align with observed values. The variability in evaluation metrics limits direct comparisons and complicates synthesising model performance from current evidence. In the future, standarised reporting frameworks and evaluation criteria must be created to enable the comparison of model performance and to improve the reproducibility, interpretability and overall quality of ML applications in MBD surveillance.

The primary challenge, therefore, is not simply a quest for higher accuracy but a push for trustworthy intelligence. The “black box” nature of complex algorithms like neural networks and some ensemble methods remains a significant barrier to adoption in public health, because there is a need to understand how and why these models arrive at certain predictions to prevent errors and bias while ensuring quality and trust [80]. This has fueled the movement toward Explainable AI (XAI), which seeks to render model decisions transparent [81]. Various methods have recently been proposed to help users interpret the predictions of complex models, like SHAP and LIME. LIME builds simple local surrogate models to show which features drive an individual prediction, while SHAP uses game-theoretic Shapley values to estimate each variable's contribution [52,59]. For MBD surveillance, the most effective path forward often lies not with the most complex model, but with the most interpretable and resource-efficient one that can be reliably deployed where it is needed most.

The increasing use DL in disease surveillance also deserves attention. While DL can better capture complex patterns from large, diverse datasets [82], ML models remain highly effective at these tasks and require less computing power, resources -such as energy- and equipment to produce good results [83]. Therefore, developing precise models that can use local data and require minimal computing resources is essential. The choice between ML and DL algorithms should ultimately depend on the surveillance goals, data types, and available resources [84]. In this review, we focused primarily on ML models, acknowledging that the regions most likely to benefit from these technologies often lack sufficient infrastructure and trained healthcare professionals to interpret and act on model outputs.

Implementing AI in surveillance and its use in the field remains limited, as issues with data quality, governance, and the need for new workforce skills prevent the smooth adoption of AI in healthcare [85]. Machine learning models in global health are frequently developed using fragmented or incomplete surveillance data that may underrepresent rural or marginalised populations. As a result, models trained on such data may systematically misclassify or underdiagnose these groups, potentially exacerbating existing health inequities [86].

Furthermore, the development of ML models often involves data generated in low- and middle-income countries but analysed and deployed in institutions in the Global North, with little local capacity building or participatory design [87].This raises concerns about data sovereignty and the equitable use of health data [88]. Addressing these issues will be critical to ensure that ML-based surveillance tools are both effective and ethically responsible.

The application of these technologies in real-life settings is essential to demonstrate their practical value. At this moment, model validation should be prioritised, as should model improvement and training on larger datasets across different locations to improve accuracy. Our analysis showed that most studies relied on internal validation techniques, like standard K-fold cross-validation or hold-out splits, without performing external validation. In the context of infectious disease forecasting, where disease is structured in both time and space, standard cross-validation may allow information to leak from the training phase into the testing phase [89].

This leakage can generate spurious correlations that high-capacity algorithms, such as Random Forest and XGBoost, readily exploit. Consequently, models may appear highly accurate during validation but fail to generalise to new data, leading to systemic overfitting. This may result in an overly optimistic view of machine learning capabilities, masking how fragile these models are when deployed in noisy, real-world surveillance scenarios. Most researchers highlighted the importance of demonstrating that ML models are effective beyond specific geographic contexts, given the diverse characteristics and interactions of parasites, vectors, hosts and their environments [62,63,90,91]. Future studies should prioritise external and prospective temporal validation using unseen data.

Another important point is data quality. Several articles revealed that data related to case incidence and vectors were often lacking in certain regions, and other articles struggled with missing values, low spatial resolutions, and inconsistent time series [56,60,68,92,93]. This is even more evident in the case of emerging arboviruses, such as Mayaro, Zika, Chikungunya, and Usutu [49,57,94,95]. Additionally, the underreporting of asymptomatic infections may also play a significant role in forecasting and early-warning ML models [49,51]. We must remember that poor input data can degrade the model's performance and interpretability, thereby increase bias and promoting under- or overfitting. Important steps to ensure data quality must start with planning and collection and pass through storage, processing, and compilation, all following specific quality control and assessment standards [96].

A secondary driver of performance overestimation identified in our review is the reliance on spatially aggregated data. Most models use data grouped by large areas -like municipalities or regions-combined with meteorological and environmental predictors. While this approach can reduce data noise and simplifies model training, it introduces ecological bias defined as the incorrect assumption that area-level correlations apply to individual-level or micro-site risk [97]. Because relationships such as the effect of temperature on mosquito traits vary substantially across microclimates, coarse spatial aggregation can overestimate local risk by up to 40% [97]. Additionally, model performance may depend more on administrative boundaries than on true epidemiological dynamics [98].

ML-based tools should be applied in public healthcare institutions, where model outputs can be translated into quicker vector control and action plans to support disease management [99]. These changes introduce additional obstacles, as governments must take an active role in developing national and international frameworks to support the regulatory approval, integration and evaluation of ML tools into disease surveillance systems. Investments in data quality, governance, transparency, assessment, and audit investigation will be necessary to integrate AI effectively into MBD surveillance. Joint work among academia, industry, and public health agencies can drive these innovations while addressing ethical and privacy concerns, ultimately improving public health operations.

The human aspect of AI and ML must not be ignored. Human expertise is essential for developing models, validating results, identifying potential errors, and interpreting findings to inform decision-making [100]. AI should be seen as supporting, not replacing, human expertise. Training health personnel in disease settings should be prioritised to enable a quicker transition from traditional surveillance to more AI-driven technologies. Mosquito surveillance using AI will also require the collaboration of interdisciplinary teams, combining expertise in pathogen and vector biology, AI model development, epidemiology, diagnostic technologies, and data science [101].

Regarding the integrated approach that includes animal, human, and environmental dimensions in surveillance, the diseases chosen in our primary search query primarily affect humans and were selected from a WHO list, which may have influenced the decision to use predictors less related to animal health in model development. In our classification, vectors were considered components of the animal domain—as arthropods when information on their biology, behaviour, or ecology was used—and of the environmental domain when their occurrence was analysed in response to ecological drivers. Our findings highlight the imbalance in the use of animal-related predictors in ML models, despite the central role animals play as reservoirs, amplifying hosts, or dead-end hosts in many transmission cycles [102,103].

When animal-related variables are used, they typically involve entomological data and, in a few cases, species and host biodiversity indices. This underrepresentation may result in missed opportunities for early detection through sentinel species, vector or wildlife surveillance, and constrains the understanding of pathogen circulation and transmission dynamics. The integration of biodiversity metrics, sentinel species, land-use changes, environmental drivers, and ecological, evolutionary, and socio-ecosystem approaches is crucial for preventing outbreaks and the emergence and reemergence of certain zoonotic pathogens.

For vector-borne disease surveillance to be truly effective, ML models must incorporate multimodal, data from humans, animals (including vectors), and the environment— while ensuring sustainability and responsible data processing. Building more integrated, multisectoral datasets could enhance the predictive power of these tools. Additionally, other data sources could be integrated into MBD surveillance, such as data from health-tracking devices, mobile apps, and social media, to monitor shifts in the population's health and subsequently alert policymakers [104].

Given the threat of human-induced climate change, shifts in transmission patterns, and changing seasonal epidemiology of MBDs, the world needs new tools to aid in disease prediction and control. ML is increasingly important for strengthening surveillance capabilities within these shifting epidemiological landscapes, providing tools for the creation of early warning systems for MBDs.

## 5 Conclusion

ML has demonstrated significant potential for supporting the surveillance of MBDs, particularly in low-resource settings, and its use will continue to grow in the coming years. With improved accuracy, efficiency, and the ability to process diverse data types, as well as various models and tasks, ML can aid in MBD surveillance and inform public health decisions. However, the success of these tools depends on the quality and availability of local data, interdisciplinary collaboration, and model validation. Future work should prioritise: 1) Prospective validation of ML models in diverse, low-resource settings; 2) The development of standardised, multi-sectoral (One Health) data repositories; 3) Implementation of ML models in real-life scenarios.

## CRediT authorship contribution statement

**Mariana Geffroy:** Writing – review & editing, Writing – original draft, Visualization, Formal analysis, Data curation, Conceptualization. **Juan Vicente Bogado Machuca:** Writing – review & editing, Investigation, Data curation. **Gerardo Suzán:** Writing – review & editing. **Fernando Esponda:** Writing – original draft. **Benjamin Roche:** Writing – review & editing, Supervision, Conceptualization.

## Declaration of generative AI and AI-assisted technologies in the manuscript preparation process

During the preparation of this work, the MG and JVBM used the RAYYAN software to aid in the systematic review, including tasks such as deduplication and article selection. After using this tool, the authors reviewed and edited the content as needed and take full responsibility for the content of the published article.

## Declaration of competing interest

The authors declare that they have no known competing financial interests or personal relationships that could have appeared to influence the work reported in this paper.

## Figures and Tables

**figs1:**
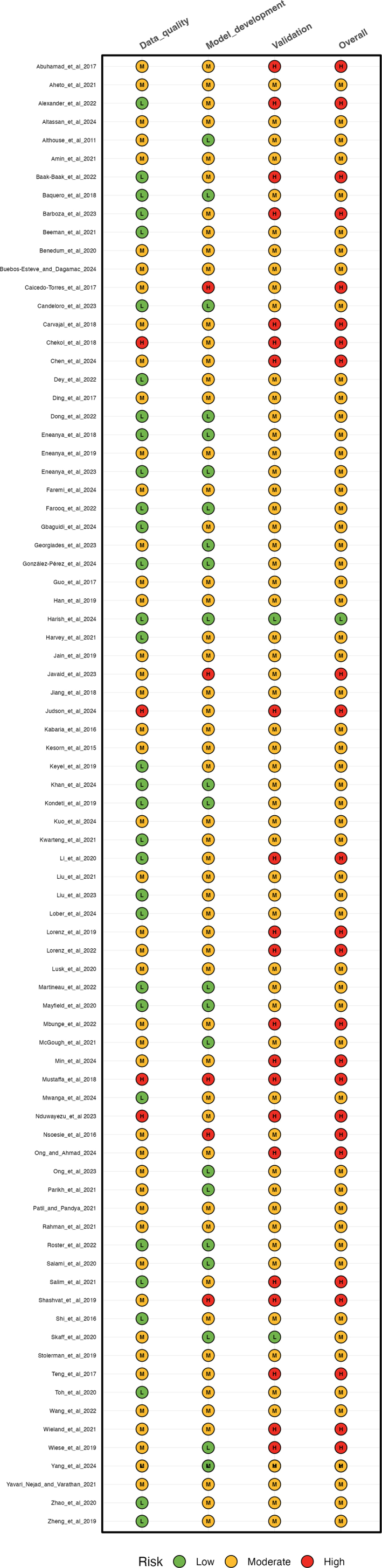


## References

[1] World Health Organization (2017). https://iris.who.int/handle/10665/259205.

[2] Ferraguti M. (2024 Sep). Mosquito species identity matters: unraveling the complex interplay in vector-borne diseases. Infect Dis.

[3] Franklinos L.H.V., Jones K.E., Redding D.W., Abubakar I. (2019 Sep). The effect of global change on mosquito-borne disease. Lancet Infect Dis.

[4] Shearer F.M., Moyes C.L., Pigott D.M., Brady O.J., Marinho F., Deshpande A. (2017 Nov 1). Global yellow fever vaccination coverage from 1970 to 2016: an adjusted retrospective analysis. Lancet Infect Dis.

[5] Lourens G.B., Ferrell D.K. (2019 Jun 1). Lymphatic filariasis. Nurs Clin.

[6] World Health Organization (2024).

[7] Ilic I., Ilic M. (2024 Mar 1). Global patterns of trends in incidence and mortality of dengue, 1990–2019: an analysis based on the global burden of disease study. Medicina.

[8] Roiz D., Pontifes P.A., Jourdain F., Diagne C., Leroy B., Vaissière A.C. (2024 Jul 10). The rising global economic costs of invasive aedes mosquitoes and Aedes-borne diseases. Sci Total Environ.

[9] Gallup J.L., Sachs J.D. (2001 Jan-Feb). The economic burden of malaria. Am J Trop Med Hyg.

[10] Haakenstad A., Harle A.C., Tsakalos G., Micah A.E., Tao T., Anjomshoa M. (2019 Jul 1). Tracking spending on malaria by source in 106 countries, 2000–16: an economic modelling study. Lancet Infect Dis.

[11] Thacker S.B., Berkelman R.L. (1988). Public health surveillance in the United States. Epidemiol Rev.

[12] Dzul-Manzanilla F., Correa-Morales F., Che-Mendoza A., Palacio-Vargas J., Sánchez-Tejeda G., González-Roldan J.F. (2021 May). Identifying urban hotspots of dengue, chikungunya, and zika transmission in Mexico to support risk stratification efforts: a spatial analysis. Lancet Planet Health.

[13] Zeller H., Marrama L., Sudre B., Bortel W.V., Warns-Petit E. (2013 Aug 1). Mosquito-borne disease surveillance by the european centre for disease prevention and control. Clin Microbiol Infection.

[14] Fournet F., Jourdain F., Bonnet E., Degroote S., Ridde V. (2018 Dec). Effective surveillance systems for vector-borne diseases in urban settings and translation of the data into action: a scoping review. Infect Dis Poverty.

[15] Dacko N.M., Nava M.R., Vitek C., Debboun M., Debboun M., Nava M.R., Rueda L.M. (2020). Mosquitoes, communities, and public health in Texas.

[16] Ramírez A.L., Van Den Hurk A.F., Meyer D.B., Ritchie S.A. (2018 Dec). Searching for the proverbial needle in a haystack: advances in mosquito-borne arbovirus surveillance. Parasites Vectors.

[17] Cabrera M., Leake J., Naranjo-Torres J., Valero N., Cabrera J.C., Rodríguez-Morales A.J. (2022 Oct 21). Dengue prediction in Latin America using machine learning and the one health perspective: a literature review. TropicalMed.

[18] Lerner H., Berg C. (2017 Sep 29). A comparison of three holistic approaches to health: one health, EcoHealth, and planetary health. Front Vet Sci.

[19] Benelli G., Duggan M.F. (2018 Jun). Management of arthropod vector data – social and ecological dynamics facing the one health perspective. Acta Trop.

[20] Braks M., Medlock J.M., Hubalek Z., Hjertqvist M., Perrin Y., Lancelot R. (2014). Vector-borne disease intelligence: strategies to deal with disease burden and threats. Front Public Health.

[21] Uchtmann N., Herrmann J.A., Hahn E.C., Beasley V.R. (2015 Jun 1). Barriers to, efforts in, and optimization of integrated one health surveillance: a review and synthesis. EcoHealth.

[22] Abbass H. (2021 Apr). Editorial: what is artificial intelligence?. IEEE Trans Artif Intell.

[23] Schwalbe N., Wahl B. (2020 May). Artificial intelligence and the future of global health. Lancet.

[24] Siddig E.E., Eltigani H.F., Ahmed A. (2023 Dec). The rise of AI: how artificial intelligence is revolutionizing infectious disease control. Ann Biomed Eng.

[25] Helm J.M., Swiergosz A.M., Haeberle H.S., Karnuta J.M., Schaffer J.L., Krebs V.E. (2020 Feb). Machine learning and artificial intelligence: definitions, applications, and future directions. Curr Rev Musculoskelet Med.

[26] Dahiya N., Gupta S., Singh S. (2022 Apr 24). A review paper on machine learning applications, advantages, and techniques. ECS Trans.

[27] Velankar M.R., Mahalle P.N., Shinde G.R., Velankar M.R., Mahalle P.N., Shinde G.R. (2024). Cognitive computing for machine thinking [Internet].

[28] Page M.J., McKenzie J.E., Bossuyt P.M., Boutron I., Hoffmann T.C., Mulrow C.D. (2021 Dec). The PRISMA 2020 statement: an updated guideline for reporting systematic reviews. Syst Rev.

[29] Rudin C. (2019 May 1). Stop explaining black box machine learning models for high stakes decisions and use interpretable models instead. Nat Mach Intell.

[30] Ouzzani M., Hammady H., Fedorowicz Z., Elmagarmid A. (2016 Dec 5). Rayyan—A web and mobile app for systematic reviews. Syst Rev.

[31] Wolff R.F., Moons K.G.M., Riley R.D., Whiting P.F., Westwood M., Collins G.S. (2019 Jan 1). PROBAST: a tool to assess the risk of bias and applicability of prediction model studies. Ann Intern Med.

[32] Fantom N.J., Serajuddin U. (2016). The World Bank's classification of countries by income. World bank Pol Res Work paper.

[33] Emmert-Streib F., Dehmer M. (2022 Sep 1). Taxonomy of machine learning paradigms: a data-centric perspective. WIREs Data Min Knowl Discovery.

[34] Bonaccorso G. (2018).

[35] Alloghani M., Al-Jumeily D., Mustafina J., Hussain A., Aljaaf A.J., Berry M.W., Mohamed A., Yap B.W. (2020). Supervised and unsupervised learning for data science [Internet].

[36] Varoquaux G., Colliot O. (2023). Evaluating machine learning models and their diagnostic value. Mach Learn Brain Disord.

[37] Soyiri I.N., Reidpath D.D. (2013 Jan 1). An overview of health forecasting. Environ Health Prev Med.

[38] Benedum C.M., Shea K.M., Jenkins H.E., Kim L.Y., Markuzon N., Reiner R.C. (2020 Oct 16). Weekly dengue forecasts in Iquitos, Peru; San Juan, Puerto Rico; and Singapore. PLoS Neglected Trop Dis.

[39] Martineau P., Behera S.K., Nonaka M., Jayanthi R., Ikeda T., Minakawa N. (2022 Aug 25). Predicting malaria outbreaks from sea surface temperature variability up to 9 months ahead in Limpopo, South Africa, using machine learning. Front Public Health.

[40] Keyel A.C., Elison Timm O., Backenson P.B., Prussing C., Quinones S., McDonough K.A., Shaman J. (2019 Jun 3). Seasonal temperatures and hydrological conditions improve the prediction of west nile virus infection rates in culex mosquitoes and human case counts in New York and Connecticut. PLoS One.

[41] Baquero O.S., Santana L.M.R., Chiaravalloti-Neto F., Shaman J. (2018 Apr 2). Dengue forecasting in São Paulo city with generalized additive models, artificial neural networks and seasonal autoregressive integrated moving average models. PLoS One.

[42] Farooq Z., Rocklöv J., Wallin J., Abiri N., Sewe M.O., Sjödin H. (2022 Jun). Artificial intelligence to predict west nile virus outbreaks with eco-climatic drivers. Lancet Reg Health - Europ.

[43] Wilson A.E., Lehmann C.U., Saleh S.N., Hanna J., Medford R.J. (2021). Social media: a new tool for outbreak surveillance. Antimicrob Stewardsh & Healthc Epidemiol.

[44] Althouse B.M., Ng Y.Y., Cummings D.A.T. (2011 Aug 2). Prediction of dengue incidence using search query surveillance. Crockett RJKent. PLoS Neglected Trop Dis.

[45] Guo P., Liu T., Zhang Q., Wang L., Xiao J., Zhang Q., Althouse B. (2017 Oct 16).

[46] Amin S., Uddin M.I., alSaeed D.H., Khan A., Adnan M., Aziz F. (2021 Jan). Early detection of seasonal outbreaks from Twitter data using machine learning approaches. Complexity.

[47] Nsoesie E.O., Flor L., Hawkins J., Maharana A., Skotnes T., Marinho F. (2016). Social media as a sentinel for disease surveillance: what does sociodemographic status have to Do with it?. PLoS Curr.

[48] Javaid M., Sarfraz M., Aftab M., Zaman Q., Rauf H., Alnowibet K. (2023 Feb 20). WebGIS-Based real-time surveillance and response system for vector-borne infectious diseases. IJERPH.

[49] Parikh N., Daughton A.R., Rosenberger W.E., Aberle D.J., Chitanvis M.E., Altherr F.M. (2021 Jan 7). Improving detection of disease Re-emergence using a web-based tool (RED alert): design and case analysis study. JMIR Public Health Surveill.

[50] Thompson P.N., Etter E.M.C. (2015).

[51] Baak-Baak C.M., Cigarroa-Toledo N., Pinto-Castillo J.F., Cetina-Trejo R.C., Torres-Chable O., Blitvich B.J. (2022 May 4). Cluster analysis of dengue morbidity and mortality in Mexico from 2007 to 2020: implications for the probable case definition. Am J Trop Med Hyg.

[52] Dong B., Khan L., Smith M., Trevino J., Zhao B., Hamer G.L. (2022 Oct 28). Spatio-temporal dynamics of three diseases caused by Aedes-borne arboviruses in Mexico. Commun Med.

[53] Kabaria C.W., Molteni F., Mandike R., Chacky F., Noor A.M., Snow R.W. (2016 Dec). Mapping intra-urban malaria risk using high resolution satellite imagery: a case study of Dar es Salaam. Int J Health Geogr.

[54] Rahman MdS., Pientong C., Zafar S., Ekalaksananan T., Paul R.E., Haque U. (2021 Dec). Mapping the spatial distribution of the Dengue vector Aedes aegypti and predicting its abundance in northeastern Thailand using machine-learning approach. One Health.

[55] Mayfield H.J., Sturrock H., Arnold B.F., Andrade-Pacheco R., Kearns T., Graves P. (2020 Nov 25). Supporting elimination of lymphatic filariasis in Samoa by predicting locations of residual infection using machine learning and geostatistics. Sci Rep.

[56] Eneanya O.A., Fronterre C., Anagbogu I., Okoronkwo C., Garske T., Cano J. (2019 Dec). Mapping the baseline prevalence of lymphatic filariasis across Nigeria. Parasites Vectors.

[57] Lorenz C., Freitas Ribeiro A., Chiaravalloti-Neto F. (2019 Oct). Mayaro virus distribution in South America. Acta Trop.

[58] Jiang D., Hao M., Ding F., Fu J., Li M. (2018 Sep). Mapping the transmission risk of zika virus using machine learning models. Acta Trop.

[59] Salami D., Sousa C.A., Martins M.D.R.O., Capinha C. (2020 Jun 16). Predicting dengue importation into Europe, using machine learning and model-agnostic methods. Sci Rep.

[60] Yang S., yang Li R., ning Yan S., yin Yang H., Cao Z you, Zhang L. (2024 Mar 20). Risk assessment of imported malaria in China: a machine learning perspective. BMC Public Health.

[61] Yan J., Gangoso L., Ruiz S., Soriguer R., Figuerola J., Martínez-de la Puente J. (2021 Aug 1). Understanding host utilization by mosquitoes: determinants, challenges and future directions. Biol Rev.

[62] Candeloro L., Ippoliti C., Iapaolo F., Monaco F., Morelli D., Cuccu R. (2020 Sep 19). Predicting WNV circulation in Italy using Earth observation data and extreme gradient boosting model. Remote Sens.

[63] Beeman S.P., Morrison A.M., Unnasch T.R., Unnasch R.S., DDP Silva (2021 Oct 8). Ensemble ecological niche modeling of West Nile virus probability in Florida. PLoS One.

[64] Judson S.D., Kenu E., Fuller T., Asiedu-Bekoe F., Biritwum-Nyarko A., Schroeder L.F., Coffee M. (2024 Oct 21). Yellow fever in Ghana: predicting emergence and ecology from historical outbreaks. PLOS Glob Publ Health.

[65] Alexander J., Wilke A.B.B., Mantero A., Vasquez C., Petrie W., Kumar N., Xue B. (2022 Dec 30). Using machine learning to understand microgeographic determinants of the Zika vector, Aedes aegypti. PLoS One.

[66] Eneanya O.A., Cano J., Dorigatti I., Anagbogu I., Okoronkwo C., Garske T. (2018 Dec). Environmental suitability for lymphatic filariasis in Nigeria. Parasites Vectors.

[67] Ding F., Fu J., Jiang D., Hao M., Lin G. (2018 Feb). Mapping the spatial distribution of Aedes aegypti and Aedes albopictus. Acta Trop.

[68] Georgiades P., Proestos Y., Lelieveld J., Erguler K. (2023 May 9). Machine learning modeling of Aedes albopictus habitat suitability in the 21st century. Insects.

[69] Arrubla-Hoyos W., Gómez J.G., De-La-Hoz-Franco E. (2024 Sep 20). Differential classification of dengue, zika, and Chikungunya using machine learning—random forest and decision tree techniques. Informatics.

[70] Mercado C.E.G., Ekapirat N., Dondorp A.M., Maude R.J. (2017 Mar 21). An assessment of national surveillance systems for malaria elimination in the Asia Pacific. Malar J.

[71] Beatty M.E., Stone A., Fitzsimons D.W., Hanna J.N., Lam S.K., Vong S. (2010 Nov 16). Best practices in dengue surveillance: a report from the Asia-Pacific and americas dengue prevention boards. PLoS Neglected Trop Dis.

[72] Organization W.H. (2025). Report on the global arbovirus surveillance and response capacity survey 2021-2022.

[73] Carabali M., Jaramillo-Ramirez G.I., Rivera V.A., Mina Possu N.J., Restrepo B.N., Zinszer K. (2021 Feb 4). Assessing the reporting of dengue, chikungunya and zika to the national surveillance system in Colombia from 2014–2017: a capture-recapture analysis accounting for misclassification of arboviral diagnostics. PLoS Neglected Trop Dis.

[74] Kading R.C., Cohnstaedt L.W., Fall K., Hamer G.L. (2020). Emergence of arboviruses in the United States: the boom and bust of funding, innovation, and capacity. Trop Med Infect Dis.

[75] Maslej N., Fattorini L., Perrault R., Gil Y., Parli V., Kariuki N. (2025).

[76] Sanabani S.S. (2025 Sep 6). Epidemiology of dengue in Brazil: recent trends and public health response. Discov Publ Health.

[77] Bangoura S., Keita A.K., Diaby M., Sidibé S., Le-Marcis F., Camara S. (2025). Arbovirus epidemics as global health imperative, Africa, 2023. Emerg Infect Dis J.

[78] Chen R.C., Dewi C., Huang S.W., Caraka R.E. (2020 Jul 23). Selecting critical features for data classification based on machine learning methods. J Big Data.

[79] Fernández-Delgado M., Cernadas E., Barro S., Amorim D. (2014). Do we need hundreds of classifiers to solve real world classification problems?. J Mach Learn Res.

[80] Quinn T.P., Jacobs S., Senadeera M., Le V., Coghlan S. (2022 Feb 1). The three ghosts of medical AI: can the black-box present deliver?. Artif Intell Med.

[81] Johannssen A., Chukhrova N. (2025 Sep 1). The crucial role of explainable artificial intelligence (XAI) in improving health care management. Health Care Manag Sci.

[82] LeCun Y., Bengio Y., Hinton G. (2015 May 1). Deep learning. Nature.

[83] Vente T., Wegmeth L., Said A., Beel J. (2024).

[84] Pichler M., Hartig F. (2023 Apr 1). Machine learning and deep learning—A review for ecologists. Methods Ecol Evol.

[85] Ige T.O. (2024).

[86] Joseph J. (2025). Algorithmic bias in public health AI: a silent threat to equity in low-resource settings. Front Public Health.

[87] Yu L., Zhai X. (2024 Sep 1). Use of artificial intelligence to address health disparities in low- and middle-income countries: a thematic analysis of ethical issues. Public Health.

[88] Qato D.M. (2022). Reflections on ‘decolonizing’big data in global health. Ann Glob Health.

[89] Cheng Y., Bai Y., Yang J., Tan X., Xu T., Cheng R. (2024 Nov 19). Analysis and prediction of infectious diseases based on spatial visualization and machine learning. Sci Rep.

[90] Kondeti P.K., Ravi K., Mutheneni S.R., Kadiri M.R., Kumaraswamy S., Vadlamani R. (2019). Applications of machine learning techniques to predict filariasis using socio-economic factors. Epidemiol Infect.

[91] Patil P.N., J U., Dharani A. (2024). 2024 international conference on integrated circuits and communication systems (ICICACS). Raichur.

[92] Caicedo-Torres W., Montes-Grajales D., Miranda-Castro W., Fennix-Agudelo M., Agudelo-Herrera N., Solano A., Ordoñez H. (2017). Advances in computing.

[93] Zhao N., Charland K., Carabali M., Nsoesie E.O., Maheu-Giroux M., Rees E., Choisy M. (2020 Sep 24). Machine learning and dengue forecasting: comparing random forests and artificial neural networks for predicting dengue burden at national and sub-national scales in Colombia. PLoS Neglected Trop Dis.

[94] Altassan K.K., Morin C.W., Hess J.J. (2024 Feb 28). Modeling the role of weather and pilgrimage variables on dengue fever incidence in Saudi Arabia. Pathogens.

[95] Chen J., Zhang Y., Zhang X., Zhang M., Yin X., Zhang L. (2024). Epidemiology and ecology of usutu virus infection and its global risk distribution. Viruses.

[96] McCord S.E., Webb N.P., Van Zee J.W., Burnett S.H., Christensen E.M., Courtright E.M. (2021 Jun 1). Provoking a cultural shift in data quality. Bioscience.

[97] Boser A., Sousa D., Larsen A., MacDonald A. (2021 Nov 18). Micro-climate to macro-risk: mapping fine scale differences in mosquito-borne disease risk using remote sensing. Environ Res Lett.

[98] Yin S., Ren C., Shi Y., Hua J., Yuan H.Y., Tian L.W. (2022). A systematic review on modeling methods and influential factors for mapping dengue-related risk in urban settings. Int J Environ Res Publ Health.

[99] Pley C., Evans M., Lowe R., Montgomery H., Yacoub S. (2021 Oct 1). Digital and technological innovation in vector-borne disease surveillance to predict, detect, and control climate-driven outbreaks. Lancet Planet Health.

[100] Villanueva-Miranda I., Xiao G., Xie Y. (2025). Artificial intelligence in early warning systems for infectious disease surveillance: a systematic review. Front Public Health.

[101] Wong F., de la Fuente-Nunez C., Collins J.J. (2023 Jul 14). Leveraging artificial intelligence in the fight against infectious diseases. Science.

[102] Figueiredo L.T.M. (2019). Human urban arboviruses can infect wild animals and jump to sylvatic maintenance cycles in South America. Front Cell Infect Microbiol.

[103] Gwee S.X.W., St John A.L., Gray G.C., Pang J. (2021 Jun 1). Animals as potential reservoirs for dengue transmission: a systematic review. One Health.

[104] Kostkova P., Saigí-Rubió F., Eguia H., Borbolla D., Verschuuren M., Hamilton C. (2021). Data and digital solutions to support surveillance strategies in the context of the COVID-19 pandemic. Front Digit Health.

[105] Husam I.S.A., Abu Bakar A., Zainudin S., Sahani M., Mohd Ali Z. (2017 Feb 28). Feature selection algorithms for Malaysian dengue outbreak detection model. JSM.

[106] Aheto J.M.K., Duah H.O., Agbadi P., Nakua E.K. (2021 Sep). A predictive model, and predictors of under-five child malaria prevalence in Ghana: how do LASSO, ridge and Elastic net regression approaches compare?. Prev Med Rep.

[107] Barboza L.A., Chou-Chen S.W., Vásquez P., García Y.E., Calvo J.G., Hidalgo H.G. (2023 Jan 13). Assessing dengue fever risk in Costa Rica by using climate variables and machine learning techniques. Hayden MH. PLoS Neglected Trop Dis.

[108] Buebos-Esteve D.E., Dagamac N.H.A. (2024 Jul). Spatiotemporal models of dengue epidemiology in the Philippines: integrating remote sensing and interpretable machine learning. Acta Trop.

[109] Carvajal T.M., Viacrusis K.M., Hernandez L.F.T., Ho H.T., Amalin D.M., Watanabe K. (2018 Dec). Machine learning methods reveal the temporal pattern of dengue incidence using meteorological factors in metropolitan Manila, Philippines. BMC Infect Dis.

[110] Chekol B.E., Hagras H. (2018). 2018 10th computer science and electronic engineering (CEEC). Colchester.

[111] Dey S.K., Rahman MdM., Howlader A., Siddiqi U.R., Uddin K.M.M., Borhan R., Bhattacharjee D. (2022 Jul 20). Prediction of dengue incidents using hospitalized patients, metrological and socio-economic data in Bangladesh: a machine learning approach. PLoS One.

[112] Eneanya O.A., Reimer L.J., Fischer P.U., Weil G.J. (2023 Sep 1). Geospatial modelling of lymphatic filariasis and malaria co-endemicity in Nigeria. Int Health.

[113] Faremi A.S., Akinnuwesi B., Mbunge E., Mashwama P., Fashoto S.G., Zenzo Ncube P. (2024). 2024 conference on information communications technology and society (ICTAS).

[114] Gbaguidi G.J., Topanou N., Filho W.L., Ketoh G.K. (2024 Feb 13). Towards an intelligent malaria outbreak warning model based intelligent malaria outbreak warning in the northern part of Benin, West Africa. BMC Public Health.

[115] González-Pérez M.I., Faulhaber B., Aranda C., Williams M., Villalonga P., Silva M. (2024 Mar 1). Field evaluation of an automated mosquito surveillance system which classifies aedes and culex mosquitoes by genus and sex. Parasites Vectors.

[116] Han B.A., Majumdar S., Calmon F.P., Glicksberg B.S., Horesh R., Kumar A. (2019 Jun). Confronting data sparsity to identify potential sources of Zika virus spillover infection among primates. Epidemics.

[117] Harish V., Colón-González F.J., Moreira F.R.R., Gibb R., Kraemer M.U.G., Davis M. (2024 May 28). Human movement and environmental barriers shape the emergence of dengue. Nat Commun.

[118] Harvey D., Valkenburg W., Amara A., Gadekallu T.R. (2021 Jun 18). Predicting malaria epidemics in Burkina Faso with machine learning. PLoS One.

[119] Kuo C.Y., Yang W.W., Su E.C.Y. (2024 Mar 20). Improving dengue fever predictions in Taiwan based on feature selection and random forests. BMC Infect Dis.

[120] Jain R., Sontisirikit S., Iamsirithaworn S., Prendinger H. (2019 Mar 21). Prediction of dengue outbreaks based on disease surveillance, meteorological and socio-economic data. BMC Infect Dis.

[121] Kesorn K., Ongruk P., Chompoosri J., Phumee A., Thavara U., Tawatsin A. (2015 May 11). Morbidity rate prediction of dengue hemorrhagic fever (DHF) using the support vector machine and the Aedes aegypti infection rate in similar climates and geographical areas. Hwang JS. PLoS One.

[122] Khan O., Ajadi J.O., Hossain M.P., Veerappampalayam Easwaramoorthy S. (2024 May 16).

[123] Kwarteng E.V.S., Andam-Akorful S.A., Kwarteng A., Asare D.C.B., Quaye-Ballard J.A., Osei F.B. (2021 Dec). Spatial variation in lymphatic filariasis risk factors of hotspot zones in Ghana. BMC Public Health.

[124] Li C., Wu X., Wang X., Yin J., Zheng A., Yang X. (2021 Jan). Ecological environment and socioeconomic factors drive long-term transmission and extreme outbreak of dengue fever in epidemic region of China. J Clean Prod.

[125] Liu K., Yin L., Zhang M., Kang M., Deng A.P., Li Q.L. (2021 Mar 25). Facilitating fine-grained intra-urban dengue forecasting by integrating urban environments measured from street-view images. Infect Dis Poverty.

[126] Liu H., Huang X., Guo X., Cheng P., Wang H., Liu L. (2023 Mar 24). Climate change and Aedes albopictus risks in China: current impact and future projection. Infect Dis Poverty.

[127] Lober L., Roster K.O., Rodrigues F.A. (2024 Oct). Forecasting infectious diseases in Brazilian cities: integrating socio-economic and geographic data from related cities through a machine learning approach. Chaos Solitons Fractals.

[128] Lorenz C., De Azevedo T.S., Chiaravalloti-Neto F. (2022 Nov 1). Impact of climate change on West Nile virus distribution in South America. Trans Roy Soc Trop Med Hyg.

[129] Lusk R., Zimmerman J., VanMaldeghem K., Kim S., Roth N.M., Lavinder J. (2020 Nov). Exploratory analysis of machine learning approaches for surveillance of zika‐associated birth defects. Birth Def Res.

[130] Mbunge E., Millham R.C., Sibiya M.N., Takavarasha S. (2022). 2022 conference on information communications technology and society (ICTAS).

[131] McGough S.F., Clemente L., Kutz J.N., Santillana M. (2021 Jun). A dynamic, ensemble learning approach to forecast dengue fever epidemic years in Brazil using weather and population susceptibility cycles. J R Soc Interface.

[132] Min K.D., Baek Y.J., Hwang K., Shin N.R., Lee S dam, Kan H. (2024). Fine-Scale spatial prediction on the risk of *Plasmodium vivax* infection in the Republic of Korea. J Kor Med Sci.

[133] Mustaffa Z., Sulaiman M.H., Emawan F., Yusof Y., Mohsin M.F.M. (2018). 2018 19th IEEE/ACIS international conference on software engineering, artificial intelligence, networking and parallel/distributed computing (SNPD).

[134] Nduwayezu G., Zhao P., Kagoyire C., Eklund L., Bizimana J.P., Pilesjo P. (2023 May 25). Understanding the spatial non-stationarity in the relationships between malaria incidence and environmental risk factors using geographically weighted random forest: a case study in Rwanda. Geospat Health.

[135] Ong S.Q., Ahmad H. (2024 Mar 1). Tracking mosquito-borne diseases via social media: a machine learning approach to topic modelling and sentiment analysis. PeerJ.

[136] Ong S.Q., Isawasan P., Ngesom A.M.M., Shahar H., Lasim A.M., Nair G. (2023 Nov 5). Predicting dengue transmission rates by comparing different machine learning models with vector indices and meteorological data. Sci Rep.

[137] Patil S., Pandya S. (2021 Nov 26). Forecasting dengue hotspots associated with variation in meteorological parameters using regression and time series models. Front Public Health.

[138] Roster K., Connaughton C., Rodrigues F.A. (2022 Sep 28). Machine-Learning–Based forecasting of dengue fever in Brazilian cities using epidemiologic and meteorological variables. Am J Epidemiol.

[139] Salim N.A.M., Wah Y.B., Reeves C., Smith M., Yaacob W.F.W., Mudin R.N. (2021 Jan 13). Prediction of dengue outbreak in Selangor Malaysia using machine learning techniques. Sci Rep.

[140] Shashvat K., Basu R., Bhondekar P., Kaur A. (2019).

[141] Shi Y., Liu X., Kok S.Y., Rajarethinam J., Liang S., Yap G. (2016 Sep). Three-month real-time dengue forecast models: an early warning system for outbreak alerts and policy decision support in Singapore. Environ Health Perspect.

[142] Skaff N.K., Cheng Q., Clemesha R.E., Collender P.A., Gershunov A., Head J.R. (1932). Thermal thresholds heighten sensitivity of West nile virus transmission to changing temperatures in coastal California. Proc Royal Soc B.

[143] Stolerman L.M., Maia P.D., Kutz J.N. (2019 Aug 8). Forecasting dengue fever in Brazil: an assessment of climate conditions. Samy AM. PLoS One.

[144] Teng Y., Bi D., Xie G., Jin Y., Huang Y., Lin B., Paul R. (2017 Jan 6).

[145] Toh K.B., Bliznyuk N., Valle D. (2021 Feb). Improving national level spatial mapping of malaria through alternative spatial and spatio-temporal models. Spat Spatio-Temp Epidemiol.

[146] Wang X., An Q., He Z., Fang W., Fuentes M. (2021 Jan).

[147] Wieland R., Kuhls K., Lentz H.H.K., Conraths F., Kampen H., Werner D. (2021 Jul). Combined climate and regional mosquito habitat model based on machine learning. Ecol Model.

[148] Wiese D., Escalante A.A., Murphy H., Henry K.A., Gutierrez-Velez V.H., Abellán P. (2019 Oct 17).

[149] Yang C., Futami K., Nihei N., Fujita R., Ogino K., Hirabayashi K., Kemenesi G. (2024 May 9).

[150] Yavari Nejad F., Varathan K.D. (2021 Dec). Identification of significant climatic risk factors and machine learning models in dengue outbreak prediction. BMC Med Inf Decis Making.

[151] Zheng X., Zhong D., He Y., Zhou G. (2019 Dec). Seasonality modeling of the distribution of Aedes albopictus in China based on climatic and environmental suitability. Infect Dis Poverty.

