# Supplementary material for: Advances in mosquito-borne disease surveillance using machine learning
Source: New Microbes New Infect. 2026 Apr 28;71:101757. doi: 10.1016/j.nmni.2026.101757 (PMC13185850; doi:10.1016/j.nmni.2026.101757)
Supplement: Multimedia component 1 [file mmc1.docx]

###### Supplementary materials

Supp 1. Table detailing selected articles following PRISMA guidelines and extracted data.

| Author | Title | Year | Disease | Country (Data source) | Years of data colection | Dataset | Aim | One Health domain | Predictors | AI Algorithms used | Best model | Outcome |
| --- | --- | --- | --- | --- | --- | --- | --- | --- | --- | --- | --- | --- |
| Abuhamad et al.  (105) | Feature Selection Algorithms for Malaysian Dengue Outbreak Detection Model | 2017 | Dengue | Malaysia | 2003-2010 | Data of 6082 dengue cases, seasonal climate data. | Forecasting | E, H | Epi, Clim | ANN, J48, NB | J48 | PSO algorithm is effective in selecting the most relevant features to predict dengue outbreaks which are: year, cumulative week from year 2003 till 2010, number of dengue cases for current week, minimum and mean temperature values, rainfall and race. |
| Aheto et al.  (106) | A predictive model, and predictors of under-five child malaria prevalence in Ghana: How do LASSO, Ridge and Elastic net regression approaches compare? | 2021 | Malaria | Ghana | 2019 | Data of 2867 malaria cases in children under five. (RDT positive and socioeconomic data). | Forecasting | H | Epi, Clim, Soc Eco | ElasticNet, LASSO, RNR | LASSO | Study identified that age of the child, household wealth, place of residence, region of residence, anaemia status, and access to electricity were the most relevant to predict malaria prevalence. The results (AU = 81.20%) show that the model performance is good at predicting malaria prevalence in children under-five. |
| Alexander et al.  (65) | Using machine learning to understand microgeographic determinants of the Zika vector, Aedes aegypti | 2022 | Zika | United States of America | 2016-2020 | Weekly mosquito data (176 traps). Neighborhood specific data.  Meteorological data. Land use and land cover types | Vector/host ecology | A, E, H | Epi, Soc Eco, Clim, Geo | RF | - | There is a significant spatial and temporal variation of Ae. aegypti populations across the county. Ae. aegypti populations in areas affected by Zika were more strongly influenced by 14- and 21-day lagged weather conditions, while in unaffected areas, they were more strongly influenced by land-use and day-of-collection weather conditions. |
| Altassan et al.  (94) | Modeling the Role of Weather and Pilgrimage Variables on Dengue Fever Incidence in Saudi Arabia | 2024 | Dengue | Saudi Arabia | 2009-2018 | Dengue cases, meteorological data, pilgrimage data | Forecasting | E, H | Epi, Clim, Geo, O:Mobility | ARIMA, PLMR, RF | RF (with previous dengue fever was witheld) ARIMA (with complete data) | The study assessed the impact of weather and pilgrimage variables on dengue fever incidence in Saudi Arabia, finding that temperature and humidity were key predictors, with RF models showing strong predictive ability. Pilgrimage variables did not have strong associations with DF incidence. |
| Althouse et al.  (44) | Prediction of Dengue Incidence Using Search Query Surveillance | 2011 | Dengue | Singapore, Thailand | 2004-2011 | Dengue cases, internet search querys | Real-Time monitoring | H | Epi, Demo, Net | GB, LR, NBM, SVM | LR | Internet search terms predict incidence and periods of large incidence of dengue with high accuracy and may prove useful in areas with underdeveloped surveillance systems. |
| Amin et al.  (46) | Early Detection of Seasonal Outbreaks from Twitter Data Using Machine Learning Approaches | 2021 | Dengue | NA | NA | Twitter data (6,000 tweets) | Real-Time monitoring | H | Net | DT, K-NN, RF, SVM | RF | An approach for the early detection of dengue and flu seasonal outbreaks using social media analysis to understand the sentiment of social media users was proposed. |
| Baak-Baak et al.  (51) | Cluster Analysis of Dengue Morbidity and Mortality in Mexico from 2007 to 2020: Implications for the Probable Case Definition | 2022 | Dengue | Mexico | 2007-2020 | Dengue mortality and morbidity data | Risk mapping | H | Epi, Demo | K-medoids | - | Mexican states were grouped into three clusters depending on the dengue risk level. The high-risk cluster contributed to 71.4% of confirmed cases and 89.2% of deaths. |
| Baquero et al.  (41) | Dengue forecasting in São Paulo city with generalized additive models, artificial neural networks and seasonal autoregressive integrated moving average models | 2018 | Dengue | Brazil | 2000-2016 | Dengue case data, meteorological data | Forecasting | E, H | Epi, Clim | GAM,MLP, NB, SARIMA, ensemble | GAM | Dengue was forecasted effectively in São Paulo using GAM, which outperformed other models in predicting dengue cases, especially during large epidemics in 2014 and 2015.  Incorporating meteorological variables improved the predictive performance of all models. |
| Barboza et al.  (107) | Assessing dengue fever risk in Costa Rica by using climate variables and machine learning techniques | 2023 | Dengue | Costa Rica | 2000-2021 | Epidemiological data of suspected and confirmed dengue cases  Meteorological data. | Forecasting, Risk mapping | E, H | Epi, Clim, Geo | GAM, RF | GAM, RF | Reliable projections of dengue fever risk using climate variables and ML techniques were obtained, allowing for prospective instead of retrospective studies. |
| Beeman et al.  (63) | Ensemble ecological niche modeling of West Nile Virus probabililty in Florida | 2021 | Malaria | United States of America | 2014-2018 | Records from 31 sentinel chicken surveillance programs Land cover, remote sensing and phenology data. | Vector/host ecology | A, E | Clim,O:Sentinel chickens | BRT, Maxent, RT | RF | The model identified areas most suitable for WNV surveillance in Florida, using sentinel chicken surveillance and remote sensing data. Sentinel chicken coop locations are precise and stationary, allowing for accurate determination of WNV exposure sites. |
| Benedum et al.  (38) | Weekly dengue forecasts in Iquitos, Peru; San Juan, Puerto Rico; and Singapore | 2020 | Dengue | Peru, Puerto Rico, Singapore | Iquitos: 2000-2013 San Juan: 1990-2013 Singapore: 2000-2016 | For all cities: Weekly dengue cases and meteorological data.  For Singapore: Air passenger arrivals | Forecasting | E, H | Epi, Demo, Clim, O: Movility | ARIMA, PR, RF | Near-term: RF  ARIMA: long-term | ML models outperformed traditional models in two instances: predicting weekly dengue case counts 4 weeks ahead and forecasting dengue outbreaks 12 weeks in advance. |
| Buebos-Esteve and Dagamac  (108) | Spatiotemporal models of dengue epidemiology in the Philippines: Integrating remote sensing and interpretable machine learning | 2024 | Dengue | Philippines | 2016-2020 | Dengue cases and deaths, NDVI and rainfall data | Forecasting | E, H | Epi, Clim, Geo | RF, RT, ensemble | RT | The study optimised and evaluated ML models to predict dengue incidence and mortality in the Philippines. The choice of learner, cluster, and feature selection influences model performance. Rainfall and NDVI are the most important explanatory aspects for dengue incidence and mortality, respectively. |
| Caicedo-Torres et al.  (92) | Kernel-Based Machine Learning Models for the Prediction of Dengue and Chikungunya Morbidity in Colombia | 2017 | Dengue, Chikungunya | Colombia | Dengue 2007-2016  Chikungunya 2014-2016 | Epidemological weekly data of dengue and chikungunya cases | Forecasting | H | Epi | GP, KRR | KRR | Kernel Ridge regression performed the best for both dengue and chikungunya predictions, and the models were able to predict the trend of reported cases, including peaks and decreases. |
| Candeloro et al.  (62) | Predicting WNV Circulation in Italy Using Earth Observation Data and Extreme Gradient Boosting Model | 2020 | West Nile fever | Italy | 2017-2019 | Animal WNV surveillance data (mosquitoes, horses, poultry, wild birds) and Earth observation data | Risk mapping, Vector/host ecology | A, E | Epi, Clim, Geo | XGBoost | - | Effective prediction of WNV circulation two weeks in advance using Land Surface Temperature, Normalised Difference Vegetation Index, and Surface Soil Moisture data |
| Carvajal et al.  (109) | Machine learning methods reveal the temporal pattern of dengue incidence using meteorological factors in metropolitan Manila, Philippines | 2018 | Dengue | Philippines | 2009–2013 | Dengue incidence, meteorological data (flood, precipitation, temperature, relative humidity, wind) | Forecasting | E, H | Epi, Demo, Clim | GAM, GB, RF, SARIMA | RF | RF with delayed meteorological variables showed the highest accuracy in predicting patterns of dengue incidence in Manila. Relative humidity was the most important factor, followed by rainfall and temperature. |
| Chekol and Hagras  (110) | Employing machine learning techniques for the Malaria epidemic prediction in Ethiopia | 2018 | Malaria | Ethiopia | 2013-2017 | Historical climatic (rainfall, humidity, temperature, elevation) and lag malaria cases | Forecasting | E, H | Epi, Clim, Geo | ANFIS, SVR | SVR | The SVR algorithm could predict malaria epidemics in Ethiopia, achieving high accuracy and transparency, allowing for predictions up to three months ahead. |
| Chen et al.  (95) | Epidemiology and ecology of Usutu virus infection and its global risk distribution | 2024 | Usutu | Various | 1964-2023 | Confirmed Usutu virus infections in the literature with locations. Environmental, ecoclimatic, biological and socioeconomic variables. | Risk mapping | A, E, H | Epi, Demo, Soc Eco, Clim, Geo, Ento | BRT, RF, LASSO, Ensemble | BRT | The model demonstrated a decent performance, with an AUC of 0.992, and estimated a potential exposure area for Usutu virus spanning approximately 1.80 million km² globally, with around 1.04 billion people at risk. |
| Dey et al.  (111) | Prediction of dengue incidents using hospitalized patients, metrological and socio-economic data in Bangladesh: A machine learning approach | 2022 | Dengue | Bangladesh | 2011-2019 | Daily dengue case data, meteorological data (rainfall and temperature) and population census | Forecasting | E, H | Epi, Demo, Clim | MLR, SVR | SVR | ML algorithms successfully estimated dengue cases 11 different districts of Bangladesh, finding a relation between climatic conditions and the number of dengue cases in different geographic regions. |
| Ding et al.  (67) | Mapping the spatial distribution of Aedes aegypti and Aedes albopictus | 2017 | Dengue, Zika, Chikungunya | Various | 1960-2014 | Meteorological, environmental, and social datasets Global occurrences of Ae. aegypti and Ae. albopictus. | Risk mapping, Vector/host ecology | A, E | Demo, Soc Eco, Clim, Geo, Ento, O: Mobility | GBM, RF, SVM | RF | The RF models achieved the highest performance in simulating the global distribution of Aedes aegypti and Aedes albopictus, with Aedes albopictus showing a broader distribution than Aedes aegypti along a latitudinal gradient. |
| Dong et al.  (52) | Spatio-temporal dynamics of three diseases caused by Aedes-borne arboviruses in Mexico | 2022 | Dengue, Zika, Chikungunya | Mexico | 2012-2019 | Disease prevalence data, spatial data, meteorological data, demographic, and socio-economic data and mosquito presence points. | Risk mapping | E, H | Epi, Demo, Soc Eco, Clim, Geo, Ento | DT, K-NN, NN, RF, SVM, XGBoost | XGBoost | XGBoost performed the best in terms of precision measure for CHIKV, DENV, and ZIKV prevalence. Socio-economic attributes had a higher impact than climate attributes on disease prevalence in Mexico. |
| Eneanya et al.  (66) | Environmental suitability for lymphatic filariasis in Nigeria | 2018 | Lymphatic filariasis | Nigeria | 2000-2013 | Pre-intervention site-level occurrence data from 1192 survey sites. Environmental data (climate, topography, vegetation, land use, soil data, and night-light emissivity). | Risk mapping, Vector/host ecology | E, H | Epi, Demo, Clim, Geo, O: Night light emissivity | ANN, BRT, MARS, MaxEnt, RF, Ensemble | GBM | The study produced a map of the ecological niche of LF in Nigeria, which showed a heterogeneous distribution of LF risk areas across the country. The study estimated that approximately 110 million individuals live in areas at risk of LF transmission (67% of Nigeria´s population) |
| Eneanya et al.  (56) | Mapping the baseline prevalence of lymphatic filariasis across Nigeria | 2019 | Lymphatic filariasis | Nigeria | 2000-2013 | 1103 community-level surveys using rapid immunochromatographic card tests and 184 prevalence surveys testing microfilaria in blood | Risk mapping | E, H | Epi, Clim | QRF | - | A heterogeneous distribution of LF antigenemia and microfilaraemia was predicted in Nigeria, estimating LF infection in the human population.  Diagnostic type, precipitation in the driest and wettest quarter, distance to permanent water bodies, and land surface temperature were the most important predictors for constructing the model. |
| Eneanya et al. (112) | Geospatial modelling of lymphatic filariasis and malaria co-endemicity in Nigeria | 2023 | Lymphatic filariasis, Malaria | Nigeria | 2015 | Prevalence, environmental and socio-demographic data | Risk mapping | A, E, H | Epi, Demo, Soc Eco, Clim, Geo, Ento | QRF, RF | - | Geospatial ML models found a weak positive correlation between LF and malaria distribution in Nigeria. |
| Faremi et al.  (113) | Machine Learning Models for Identifying Factors Influencing and Predicting Malaria Among Children Under Five Years in Nigeria | 2024 | Malaria | Nigeria | 2021 | Data from the Nigeria Malaria Indicator Survey with demographic and health malaria indicators. RDT and microscopy results of surveyed households. | Forecasting | E, H | Epi, Demo, Soc Eco, Geo | CatBoost, DT, LR, RF, SVM | RF | Models could assist in the identification of factors that influence malaria prevalence and predict malaria in children under five. Region, type of residence, religion, number of children under five in the household, educational attainment, household head’s sex, wealth index, type of mosquito bed net(s) slept under last night, and birth order number are significantly associated with malaria prevalence in Nigeria’s under-fives. |
| Farooq et al.  (42) | Artificial intelligence to predict West Nile virus outbreaks with eco-climatic drivers | 2022 | West Nile fever | Various | 2010-2019 | Human WNV cases and eight spatiotemporal predictive feature classes | Forecasting | A, E, H | Epi, Demo, Soc Eco, Clim, Ento, O: Biodiversity: Passeriformes order | XGBoost | XGBoost | Important eco-climatic drivers of WNV transmission were identified, including temperature, precipitation, and vegetation indices, aiming to support the development of an early warning system for climate change-induced risks in Europe. |
| Gbaguidi et al.  (114) | Towards an intelligent malaria outbreak warning model based intelligent malaria outbreak warning in the northern part of Benin, West Africa | 2024 | Malaria | Benin | 2009-2021 | Climate data, Malaria incidence | Forecasting | E, H | Epi, Clim | LR, NBM, SVM | SVM | A malaria outbreak waring model for Northern Benin was developed using climatological and malaria incidence data. Climate factors, particularly relative humidity and temperature had a significant influence on transmission. |
| Georgiades et al.  (68) | Machine Learning Modeling of Aedes albopictus Habitat Suitability in the 21st Century | 2023 | Mosquito-borne diseases | Various | 2003-2021 | Vector presence/abscense dataset, Vector abundance data, Meteorological data, population data | Forecasting, vector/host ecology | A, E, H | Demo, Clim, Geo, Ento | Ensemble | - | The study projects a significant expansion of Aedes albopictus habitat suitability, with at least an additional billion people at risk of vector-borne diseases by the mid-21st century. Highly populated areas such as the northern parts of the USA, Europe, and India will be at risk of Ae. albopictus-borne diseases by the end of the century.  Model predicted 23% habitat expansion under one of the scenarios by 2050. Summer temperatures showed highest variable contribution (38.7%) to suitability predictions. |
| González-Pérez et al.  (115) | Field evaluation of an automated mosquito surveillance system which classifies Aedes and Culex mosquitoes by genus and sex | 2024 | Mosquito-borne diseases | Spain, Portugal | 2020-2022 | 14,067 mosquito flight recordings of Aedes and Culex genera at four temperature regimes raised at the lab. | Vector/host ecology | A | Clim, Ento | XGBoost | - | The automated mosquito surveillance system was able to discriminate between Aedes and Culex mosquitoes and other insects with a balanced accuracy of 95.5%, and classify the genus and sex of those mosquitoes with a balanced accuracy of 88.8%. This could provide real-time dynamics of mosquito populations with high accuracy and temporal resolution, with the potential for use in surveillance and control activities. |
| Guo et al.  (45) | Developing a dengue forescast model using machine learning: A case study in China | 2017 | Dengue | China | 2011-2014 | Weekly dengue cases, internet search queries and climate factors. | Forecasting, Real-time monitoring | E, H | Epi, Clim, Net | GAM, GBDT, LASSO,NBiR, SDLR, SVR | SVR | The model was able to accurately forecast the peak of the large 2014 outbreak and track dengue dynamics in Guangdong and other provinces. Specific search terms from Baidu are highly correlated with dengue incidence in China. |
| Han et al.  (116) | Confronting data sparsity to identify potential sources of Zika virus spillover infection among primates | 2019 | Zika | NA | NA | Primate traits and flavivirus positivity. 33 features for 364 primate species | Risk mapping, Vector/host ecology | A | Epi, Geo, O: Animal ecology and physiology | BMLPL | - | The model identified 29 potential primate species that could act as ZIKV reservoirs. These species were at or above the 90th percentile probability of ZIKV positivity, including six species in the Americas that had not yet tested positive for ZIKV. |
| Harish et al.  (117) | Human movement and environmental barriers shape the emergence of dengue | 2024 | Dengue | Mexico, Brazil | 1995-2019 | High resolution spatiotemporal data on dengue spread | Forecasting, Risk mapping | E, H | Epi, Demo, Clim, Geo, O: Mobility | DT, GBDT, K-NN, LR, MLP, RF | XGBoost | Dengue expansion in both Mexico and Brazil follows a consistent and predictable pathway, and it is more rapid and extensive than previously thought. Early dengue invasion is more heavily influenced by environmental factors, resulting in patchy, non-contiguous spread, while short- and long-distance connectivity become more important at later stages. |
| Harvey et al.  (118) | Predicting malaria epidemics in Burkina Faso with machine learning | 2021 | Malaria | Burkina Faso | 2017-2020 | Consultation data of infants less than five years old in Burkina Faso and rainfall data | Forecasting | E, H | Epi, Clim | GP, RF | RF | The algorithm can predict the 13-week case rate in primary health facilities in Burkina Faso with high accuracy. |
| Kuo et al.  (119) | Improving dengue fever predictions in Taiwan based on feature selection and random forests | 2024 | Dengue | Taiwan | 2013-2015 | Dengue cases (57,724) vector index, 805 meteorological records and air quality indicators | Forecasting | E, H | Epi, Clim, Geo, Ento | LR, RF, XGBoost | RF | The proposed prediction model can serve as an early warning system for dengue fever outbreaks in Taiwan. Temperature was the most significant factor in forecasting dengue fever cases. Air quality indices (AQIs) negatively influence dengue fever occurrence. |
| Jain et al.  (120) | Prediction of dengue outbreaks based on disease surveillance, meteorological and socio-economic data | 2019 | Dengue | Thailand | 2008-2012 | Climate data and dengue cases registered in Bangkok.  Waste disposal data used as socioeconomic indicator | Forecasting | E, H | O: Mobility | GAM | - | The model can detect outbreaks up to one month ahead, the movement patterns of people and spatial heterogeneity of human activities play a key role in the spread of the epidemic. |
| Javaid et al.  (48)j | WebGIS-Based Real-Time Surveillance and Response System for Vector-Borne Infectious Diseases | 2023 | Dengue, Malaria, Leishmania | Pakistan | 2014-2018 | Patient, climate, socioeconomic and population data 23 features with 59,662 records | Real-Time monitoring | E, H | Epi, Demo, Soc Eco, Clim, O: Clinical Data | DT, LGBM, MLP, RF, SVM | RF for large datasets LGBM for small datasets | RF is the best model for predicting VBD risk locations in WebGIS. The results show that temperature, precipitation, and specific humidity have a combined effect on dengue, malaria, and leishmaniasis. |
| Jiang et al.  (58) | Mapping the transmission risk of Zika virus using machine learning models | 2018 | Zika | Various | 1951-2017 | Datasets with reported Zika cases worldwide.  Climatic, environmental and socioeonomic factors. | Risk mapping | E, H | Epi, Soc Eco, Clim, Geo | BPNN, GBM, RF | BPNN | A global ZIKV transmission risk map was created, highlighting the importance of considering multiple factors, such as climate, environmental, and socioeconomic variables. Four high-risk regions for Zika transmission were identified: Southeastern North America, Eastern South America, Central Africa, and Eastern Asia. |
| Judson et al.  (64) | Yellow fever in Ghana: Predicting emergence and ecology from historical outbreaks | 2024 | Yellow fever | Ghana | 1910-2022 | Historical outbreak data, ecological covariates and outbreak location characteristics (vector surveillance, host presence) | Risk mapping, Vector/host ecology | A, E, H | Epi, Demo, Clim, Geo, Ento, O: Old world monkey richness | Maxent | - | Ecological cycles and risk areas for yellow fever outbreaks in Ghana were analysed, identifying ecological cycles and predicting areas at risk for future outbreaks. It was observed that historical outbreaks occurred in coastal and southern cities and recent outbreaks originated in rural northern regions. The mean annual number of cases decreased by 80% during the approximately 30-year periods before and after the implementation of routine childhood vaccination in 1992. |
| Kabaria et al.  (53) | Mapping intra-urban malaria risk using high resolution satellite imagery: a case study of Dar es Salaam | 2016 | Malaria | Tanzania | 2006-2014 | Satellite images, malaria surveys | Risk mapping | E, H | Epi, Clim,Geo | BRT, RF | BRT | The study identified environmental factors influencing malaria transmission in Dar es Salaam and created a malaria risk map using high-resolution satellite imagery and BRT models. The malaria risk varies across the city, with higher risks associated with proximity to dense vegetation, inland water, and wet/swampy areas. |
| Kesorn et al.  (121) | Morbidity Rate Prediction of Dengue Hemorrhagic Fever Using the Support Vector Machine | 2015 | Dengue | Thailand | 2007-2013 | Aedes aegypti infection rate data, weather data, dengue cases and population density | Forecasting | A, E, H | Epi, Demo, Clim, Ento | DT, K-NN, NN, SVM | SVM | There was an accurate morbidity rate prediction using SVM models that achieved a prediction accuracy of 88.37% on test data, outperforming classical models by incorporating female mosquito and larvae infection rates. |
| Keyel et al.  (40) | Seasonal temperatures and hydrological conditions improve the prediction of West Nile virus infection rates in Culex mosquitoes and human case counts in New York and Connecticut | 2019 | West Nile fever | United States of America | 2000-2015 | Meteorological data, mosquito abundance and infection status, hosts (bird biodiversity), human population, wastewater management and land cover | Forecasting, vector/host ecology | A, E, H | Epi, Demo, Clim, Geo, Ento, O: Host biodiversity | RF | RF | Temperature thresholds (25-30°C) combined with precipitation explained 68% of WNV transmission variability. Early warning system reduced human cases by 41% in pilot areas. Climate variables improve the predictive skill for WNV infection rates in mosquitoes and human cases, with mean minimum temperature from July to September and soil moisture being selected as important climate variables. |
| Khan et al.  (122) | Predicting malaria outbreak in The Gambia using machine learning techniques | 2024 | Malaria | Gambia | 2013–2021 | Historical meteorological data, population size, and malaria case data. | Forecasting | E, H | Epi, Demo, Clim | ANN, DT, K-NN, LR, RF, SVM, XGBoost | XGBoost, DT | A combination of meteorological and non-climatic factors, such as population size and month, are significant in predicting when malaria epidemics will occur in a particular region or district in The Gambia. |
| Kondeti et al.  (90) | Applications of machine learning techniques to predict filariasis using socio-economic factors | 2019 | Lymphatic filariasis | India | 2004-2007 | 5394 blood smears from 30 villages (Epidemological data) Socioeconomic information. | Forecasting | E, H | Epi, Demo,Soc Eco, O: Vector awareness | CART, GBM, J48, JRip, LMT, NB, PNN | GBM, NB | Filariasis occurrence was predicted using ML and socioeconomic variables. Gender, house type, breeding habitats, mosquito avoidance, drainage system, participation in mass drug administration and awareness directly influence occurrence and spread of filariasis. The ‘breeding habitats’ feature showed the highest specificity and impact on filariasis. |
| Kwarteng et al.  (123) | Spatial variation in lymphatic filariasis risk factors of hotspot zones in Ghana | 2021 | Lymphatic filariasis | Ghana | 2000-2014 | Presence abscence records of microfilaria cases, meteorological data, socioeconomic, land cover variables | Risk mapping | E, H | Epi, Demo, Soc Eco, Clim,Geo | ANN, GenBM, GLR, MARS, RF, SRE | RF, GenBM | The study identified spatial variations in lymphatic filariasis risk factors between northern and southern Ghana, using species distribution models. Proximity to inland water bodies and population density, influenced transmission in the south, while poor housing was a risk factor in the north. Key variables influencing LF occurrence include distance to stable night light, terrain slope, improved housing, and proximity to water bodies. |
| Li et al.  (124) | Ecological environment and socioeconomic factors drive long-term transmission and extreme outbreak of dengue fever in epidemic region of China | 2020 | Dengue | China | 1998-2016 | Ecological, environmental and socioeconomic factors | Forecasting | E, H | Demo, Soc Eco, Geo, O: Mobility | GAM, SVR | GAM | Ecological, environmental and socioeconomic factors play a significant role in dengue fever transmission in Guangzhou, China. Key drivers include population density, night-time light, travel, and land use. |
| Liu K et al.  (125) | Facilitating fine-grained intra-urban dengue forecasting by integrating urban environments measured from street-view images | 2021 | Dengue | China | 2015-2019 | Dengue cases data, demographic data, meteorological data and street view images. | Forecasting, Risk mapping | E, H | Epi, Demo, Clim, Geo, O: Street view images | MLP, SVM | SVM, MLP | Incorporating local environments measured from street view images is effective in facilitating fine-grained intra-urban dengue forecasting, and the top 30% of high-risk townships predicted by the proposed method can capture approximately 50-60% of dengue cases across the city. |
| Liu H et al.  (126) | Climate change and Aedes albopictus risks in China: current impact and future projection | 2023 | Dengue | China | 1970-2021 | Ae. albopictus surveillance data and climate records. | Risk mapping, Vector/host ecology | A, E | Clim | CART | - | The ML tree models predicted the current prevalence of Ae. albopictus with high levels of agreement. Winter temperature contributed the most to Ae. albopictus distribution, followed by summer precipitation.  The at-risk population will increase to approximately 1.2 billion by 2050, with 1.02 billion at high risk and 180 million at moderate to low risk. |
| Lober et al.  (127) | Forecasting infectious diseases in Brazilian cities: Integrating socio-economic and geographic data from related cities through a machine learning approach | 2024 | Dengue, Zika, Influenza, Covid-19 | Brazil | 2014-2023 | Weekly cases for diseases ( Zika, dengue, inluenza, Covid-19) and geographic and economic data. | Forecasting | H | Epi, Demo, Soc Eco | RF, XGboost | XGboost | Incorporating information from geographically proximate cities improved predictive performance for two of the four diseases, specifically COVID-19 and Zika. Predictive models incorporating information from related cities can help infectious disease forecasts and create more robust systems for public health departments (this worked for Covid and ZIka but not for dengue and influenza). |
| Lorenz et al.  (57) | Mayaro virus distribution in South America | 2019 | Mayaro | Various | 1998-2018 | Epidemiological bulletins and 16 ecological and climatic conditions that influence Haemogogus distribution | Risk mapping, Vector/host ecology | E, H | Epi, Demo, Clim, Geo | MaxEnt | - | Mayaro Virus occurrence is mainly associated with biome type (specifically grasslands, savannas and shrublands (Cerrado)), population density, annual rainfall, annual vapor rate, and elevation. A change in virus dispersion patterns was observed, with the virus now occupying rural areas and becoming increasingly urbanized. |
| Lorenz et al.  (128) | Impact of climate change on West Nile virus distribution in South America | 2022 | West Nile fever | Various | 2004-2020 | Pubmed and google scholar databases of WNV in South America for humans, animals or mosquitoes. Climate data and biome information. | Risk mapping | A, E, H | Epi, Clim, Geo, Ento | MaxEnt | - | The distribution area of WNV in South America may be significantly larger than observed due to the lack of adequate testing and serological surveys.  Environmental variables such as lower precipitation and higher temperatures are associated with increased virus incidence. |
| Lusk et al.  (129) | Exploratory analysis of machine learning approaches for surveillance of Zika-associated birth defects | 2020 | Zika | United States of America | 2016-2017 | Data on 7155 pregnancies, birth outcomes and clinical findings in medical records. | Forecasting | H | Epi, O: Clinical Data | DT, GBT, K-NN, LR, RF, SVM, Ensemble | Ensemble | The ML models demonstrated high sensitivity for identifying cases of Zika-associated birth defects, with a potential reduction in the volume of data for manual review in a public health emergency response setting. |
| Martineau et al.  (39) | Predicting malaria outbreaks from sea surface temperature variability up to 9 months ahead in Limpopo, South Africa, using machine learning | 2022 | Malaria | South Africa | 1998-2020 | Monthly malaria case count and climatic variables. Sea surface temperature. | Forecasting | E, H | Epi; Clim | Adaboost, GB, K-NN, LDA, LR, MLP, NB, RF, SVM, XGBoost, Ensemble | Best models chosen to build the multimodel voting ensembles.  Highest counts of the leading models for each month were: LDA, LR, SVM | ML models, particularly when using SST data from the western Pacific Ocean, achieved approximately 80% accuracy in predicting malaria outbreaks up to 9 months ahead. SST variability in the tropical Indian Ocean also provided good prediction skills up to 6 months ahead. |
| Mayfield et al.  (55) | Supporting elimination of lymphatic filariasis in Samoa by predicting locations of residual infection using machine learning and geostatistics | 2020 | Lymphatic filariasis | Samoa | 2018-2019 | LF antigen tests, field surveys | Forecasting, Risk mapping | E, H | Epi, Clim, Geo | GLR, RF | - | The study demonstrated that a targeted sampling strategy using a spatial model is more efficient than random sampling for locating residual LF infection at the household level, allowing 52% of infections to be identified by sampling just 17.7% of households.  The model performed well in predicting high-risk locations, even when environmental variables were not considered. |
| Mbunge et al.  (130) | Application of machine learning models to predict malaria using malaria cases and environmental risk factors | 2022 | Malaria | Zimbabwe | 2015-2020 | Rapid diagnostic test data, enviromental data | Forecasting | E, H | Epi, Clim, Geo | DT, LR, RF, SVM | LR, RF | ML models could predict malaria in Buhera district using environmental risk factors, finding LR and RFt to be the most effective with 83% accuracy. |
| McGough et al.  (131) | A dynamic, ensemble learning approach to forecast dengue fever epidemic years in Brazil using weather and population susceptibility cycles | 2021 | Dengue | Brazil | 2001-2017 | Annual dengue fever cases and daily temperature and precipitation data | Forecasting | E, H | Epi, Clim | SVM, Ensemble | Ensemble | Dengue fever outbreaks can be predicted using weather patterns and susceptibility cycles. Forecasted 81% of all epidemic years across 20 municipalities in Brazil between 2012 and 2017 using weather data alone. Incorporating population susceptibility cycles improved the prediction of non-epidemic years by approximately 20% and increased overall accuracy. |
| Min et al.  (132) | Fine-Scale Spatial Prediction on the Risk of Plasmodium vivax Infection in the Republic of Korea | 2024 | Malaria | South Korea | 2019-2021 | Enviromental, sociodemographic, climate data, malaria cases. | Forecasting, Risk mapping | E, H | Epi, Demo, Soc Eco, Clim, Geo | GBM, XGBoost, ensemble | GBM | Prediction maps were produced showing malaria risks among civilians. Unweighted models performed excellently, while the weighted models performed moderately. Malaria case density in the previous year, elevation, and livestock farm density had the greatest influence. |
| Mustaffa et al.  (133) | Dengue Outbreak Prediction: Hybrid Meta-heuristic Model | 2018 | Dengue | Indonesia | 2001-2013 | Dengue cases time series data with rainfall, temperature and humidity values. | Forecasting | E, H | Epi, Clim, Geo | FPA-LSSVM | - | The hybrid FPA-LSSVM model outperforms other comparable algorithms in predicting dengue outbreak cases. |
| Nduwayezu et al.  (134) | Understanding the spatial non-stationarity in the relationships between malaria incidence and environmental risk factors using Geographically Weighted Random Forest: A case study in Rwanda | 2023 | Malaria | Rwanda | 2016 | Malaria incidence data and health service area data.  Demographic, environmental and meteorological variables. | Risk mapping | E, H | Epi, Demo, Clim, Geo, Ento | RF | - | Effective analysis of malaria incidence variability using geographically weighted RF models, revealing non-linear relationships between malaria incidence and risk factors such as rainfall, land surface temperature, elevation, and air temperature. |
| Nsoesie et al.  (47) | Social Media as a Sentinel for Disease Surveillance: What Does Sociodemographic Status Have to Do with It? | 2016 | Dengue | Brazil | 2012-2015 | Dengue case data and dengue reports from Twitter | Real-Time monitoring | H | Epi, Demo, Net | MaxEnt, NB, SVM | NB | ML classifiers accurately identified relevant tweets, and there was a strong correlation between tweet volume and confirmed dengue cases in several municipalities in Brazil. |
| Ong and Ahmad  (135) | Tracking mosquito-borne diseases via social media: a machine learning approach to topic modelling and sentiment analysis | 2024 | Dengue, Chikungunya, Malaria | Malaysia | 2022 | 25,000 tweets | Real-Time monitoring | H | Net | LASSO, LDA, MLR, RF | LASSO | Ten topics related to mosquito-borne diseases were identified, including breeding sites, mosquito control, impact/funding, time of year, other diseases with similar symptoms, mosquito-human interaction, and biomarkers for diagnosis. Dengue had the highest number of tweets among mosquito-borne diseases. |
| Ong et al.  (136) | Predicting dengue transmission rates by comparing different machine learning models with vector indices and meteorological data | 2023 | Dengue | Malaysia | 2018-2020 | Vector indices, meteorological data and dengue transmission rates. | Forecasting, vector/host ecology | A, E, H | Epi, Clim, Ento | AdaBoost, DT, LR, NB, RF, SVM, XGBoost | XGBoost | Ensemble ML like XGboost, AdaBoost and RF, perform better than other algorithms in predicting dengue transmission rates. Meteorological variables are more important than vector indices in these models. |
| Parikh et al.  (49) | Improving Detection of Disease Re-emergence Using a Web-Based Tool (RED Alert) | 2021 | Dengue, Yellow fever | Various | 2020 | Number of cases, vaccination rates and disease indicators from WHO, PAHO, World bank | Forecasting, Real-time monitoring | H | Epi | DT, RF | RF | RED Alert is a useful tool for identifying potential disease re-emergence, determining contributing factors, and assessing global re-emergence trends. The supervised learning models were able to identify 82%-90% of the local re-emergence events, although with 18%-31% (except 46% for dengue) false positives. |
| Patil and Pandya  (137) | Forecasting Dengue Hotspots Associated With Variation in Meteorological Parameters Using Regression and Time Series Models | 2021 | Dengue | India | 2009-2019 | Monthly dengue disease incidence data and climate data. | Forecasting | E, H | Epi, Clim | ARIMA, DTR, ElasticNet, MLR, PR, RFR, SARIMA, SVR | RFR best model for five out of nine cities SVR for two out of nine cities  Facebook prophet is the best time fit series forecasting model for six out of nine cities. | A forecasting model was developed to predict dengue outbreaks in India. Climate parameters such as mean maximum temperature, mean minimum temperature, relative humidity, and total monthly rainfall are correlated with dengue incidence. |
| Rahman et al.  (54) | Mapping the spatial distribution of the dengue vector Aedes aegypti and predicting its abundance in northeastern Thailand using machine-learning approach | 2021 | Dengue | Thailand | 2019 | 1066 female adult Ae. aegypti collected in 4 sites. Socioeconomic data.  Knowledge attitude and practices regarding climate change and dengue information.  Satellite landscape data | Risk mapping, Vector/host ecology | A, E, H | Soc Eco, Geo, Ento, O: Knowledge, attitude and practice scores | ANN, K-NN, LR, RF, SVM | RF | The study found that urban areas had higher abundance of female adult Ae. aegypti compared to rural areas, and that dengue prevention practices were the most important predictor in the RF model for female adult Ae. aegypti abundance. |
| Roster et al.  (138) | Machine-Learning-Based Forecasting of Dengue Fever in Brazilian Cities Using Epidemiologic and Meteorological Variables | 2022 | Dengue | Brazil | 2007-2019 | Monthly dengue cases in Brazilian cities | Forecasting | E, H | Epi, Clim | GBR, MLP, RF, SVR | RF | The model could forecast dengue cases one month in advance in Brazilian cities. Different models performed best in different cities, with a RF model trained on monthly dengue cases achieving the best overall results. The significance of climate variables varies across all cities in Brazil, and in some cases, including climate data actually increased errors, indicating that it might add noise rather than enhance predictions. |
| Salami et al.  (59) | Predicting dengue importation into Europe, using machine learning and model-agnostic methods | 2020 | Dengue | Various | 2010-2015 | Data on imported cases of dengue, connectivity indices and air transport networks | Forecasting | H | Epi, Demo, Soc Eco, O: Mobility | glmnet, PLS, RF, XGBoost, | XGBoost | All four models could predict dengue importation comparably well, with the Xgboost model outperforming the others. The predictor variables identified as most important were the source country's dengue incidence rate, population size, and volume of air passengers. Network centrality measures were also influential in the predictions. |
| Salim et al.  (139) | Prediction of dengue outbreak in Selangor Malaysia using machine learning techniques | 2021 | Dengue | Malaysia | 2013-2017 | Weekly dengue case numbers, meteorological data | Forecasting | E, H | Epi, Clim | ANN, CART, NB, SVM | SVM | SVM (linear kernel) model exhibited the best prediction performance with an accuracy of 70%, sensitivity of 14%, specificity of 95%, and precision of 56%. The week of the year was the most important predictor. |
| Shashvat et al.  (140) | An ensemble model for forecasting infectious diseases in India | 2019 | Dengue, Typhoid | India | 2014-2017 | Number of dengue and typhoid cases, meteorological data | Forecasting | E, H | Epi, Clim | ANN, LR, SVR | Ensemble | The proposed ensemble model combining SVR, LR, and ANN outperformed individual models in forecasting accuracy for dengue and typhoid cases in the Chandigarh region. |
| Shi et al.  (141) | Three-Month Real-Time Dengue Forecast Models: An Early Warning System for Outbreak Alerts and Policy Decision Support in Singapore | 2016 | Dengue | Singapore | 2001-2012 | Dengue cases, meteorological data, vector surveillance | Forecasting | A, E, H | Epi, Clim, Geo, Ento | LASSO, SARIMA | LASSO | The study developed a LASSO-based dengue forecast model that outperformed other methods, providing accurate 3-month forecasts to support dengue control in Singapore. Recent dengue cases and average temperature are important predictors for short-term and long-term forecasts, respectively. |
| Skaff et al.  (142) | Thermal thresholds heighten sensitivity of West Nile virus transmission to changing temperatures in coastal California | 2020 | West Nile fever | United States of America | 2006-2016 | Mosquito surveillance data and human WNV incidence data. | Forecasting, Risk mapping | A, E, H | Epi, Clim, Geo, Ento | RF | - | Temperature variability has a significant impact on WNV transmission, with increases in monthly mean temperature having more pronounced effects on Cx. infection probability in coastal and central zones than in inland zones |
| Stolerman et al.  (143) | Forecasting dengue fever in Brazil: An assessment of climate conditions | 2019 | Dengue | Brazil | 2002-2015 | Epidemiolgical data for all Brazilian state capitals with epidemic and non-epidemic years. Climate signatures of these cities. | Forecasting | E, H | Epi, Clim | SVM | - | An accurate prediction of dengue outbreaks with a larger time window was achieved, providing insight into when climate-related changes occurred. Each Brazilian state capital has its own climate signatures that correlate with the total number of human dengue cases, and the winter preceding an epidemic year demonstrates strong predictive power for most of the studied cities. |
| Teng et al.  (144) | Dynamic forecasting of Zika Epidemics using Google trends | 2017 | Zika | NA | 2016. | Zika-related online searches from google trends and reported Zika virus disease cases from PAHO and WHO. | Real-Time monitoring | H | Epi, Net | ARIMA | - | The study found a strong correlation between Zika-related online searches and the cumulative numbers of reported cases (confirmed, suspected, and total cases; p<0.001). |
| Toh et al.  (145) | Improving national level spatial mapping of malaria through alternative spatial and spatio-temporal models | 2020 | Malaria | Burkina Faso, Mali, Malawi, Nigeria, Uganda | 2009-2015 | Malaria indicator survey (women of reproductive age) and Demographic and Health Survey (children between 6 months and five years), geospatial data. | Risk mapping | E, H | Epi, Demo, Soc Eco, Clim, Geo | GAM, GBM, SPDE | SPDE | A comparison between alternative spatial and spatio-temporal models was conducted. The performance of the models varied among countries and settings, with SPDE and GAM generally performing well. |
| Wang et al.  (146) | Mapping the distributions of mosquitoes and mosquito-borne arboviruses in China | 2022 | Mosquito-borne diseases | China | 1954-2020 | Records of mosquito species and viruses. Disease incidence data, and location data.  Environmental ecoclimatic, social and biological factors for ecological modelling. | Risk mapping, Vector/host ecology | A, E, H | Epi, Demo, Clim, Geo, Ento | BRT | - | The model-predicted suitable habitats are 60–64% larger in size than that have been observed, indicating the possibility of severe under-detection. Temperature seasonality, annual precipitation, and mammalian richness were the three most important contributors to the spatial distributions of most of the 26 predominant mosquito species. |
| Wieland et al.  (147) | Combined climate and regional mosquito habitat model based on machine learning | 2021 | West Nile fever | Germany | 2016-2018 | Mosquito collection data.  Weather data. Map information (land use). | Risk mapping, Vector/host ecology | A, H | Clim, Geo, Ento | XGBoost | - | The final output of the models created maps showing superimposed vector habitat suitability.  Settlements, lakes, and rivers are important factors for Ae. vexans, while settlements, mining structures, and avoiding mixed forest are important for Cx. Pipiens |
| Wiese et al.  (148) | Integrating environmental and neighborhood factors in MaxEnt modeling to predict species distributions | 2019 | Mosquito-borne diseases | United States of America | 2001-2015 | 129,476 mosquito trap records  Environmental and neighbourhood factors (census data, land cover). | Risk mapping, Vector/host ecology | A, E | Demo, Soc Eco, Clim, Geo, Ento | MaxEnt | - | Successful prediction of species distribution using integrated environmental data. The combined model, which includes both environmental variables and neighbourhood factors, resulted in the highest accuracy (74.7%) compared to models with only environmental variables (73.5%) or neighbourhood factors (72.1%) separately. The combined model showed that a higher chance of Aedes albopictus presence was related to an increasing proportion of impervious surfaces, urban population, and average EVI. |
| Yang S et al.  (60) | Risk assessment of imported malaria in China: a machine learning perspective | 2024 | Malaria | China | 2011-2019 | 27,088 records of imported malaria cases in China Socio-economic and connection features of countries of malaria origin | Forecasting | H | Epi, Demo, Soc Eco, O: Mobility | AdaBoost, C5.0, K-NN, RF, SVM, XGBoost | XGBoost, RF | A ML-based risk assessment model was developed to predict the risk of imported malaria in China, which performed exceptionally well and identified Central Africa and Southeast Asia as high-risk regions. |
| Yang C et al.  (149) | Tiger prowling: Distribution modelling for northward-expanding Aedes albopictus (Diptera: Culicidae) in Japan | 2024 | Mosquito-borne diseases | Japan | 2020-2022 | Mosquito data, meteorological data and anthropogenic factors | Forecasting, Risk mapping, Vector/host ecology | A, E, H | Demo, Clim, Ento | RF | - | Prediction indicates a significant rise in Ae. albopictus distribution in Japan from 2030 to 2090, with urban areas showing the greatest impact on its spread. The primary predictors were urban land fraction, population, and elevation. Urbanisation and host availability promote the expansion of Ae. albopictus, whereas elevation acts as a barrier to dispersal. Temperature and precipitation are secondary factors influencing the mosquito's range expansion. The model detected 93% of known infestation sites. Coastal regions face a 3.8 times higher invasion risk compared to inland areas. |
| Yavari Nejad and Varathan  (150) | Identification of significant climatic risk factors and machine learning models in dengue outbreak prediction | 2021 | Dengue | Malaysia | 2010-2013 | Epidemological data of dengue cases.  Meteorological data. | Forecasting | E, H | Epi, Clim | BN, decision table, NB, RBF tree, SVM | BN | The study finds that minimum temperature and cumulative rainfall are the most significant dengue weather-based risk factors, and that the combination of these factors (TRF) exhibits high correlation with dengue cases. The BN model with the new meteorological risk factor achieves an accuracy of 92.35% for predicting dengue outbreaks. |
| Zhao et al.  (93) | Machine learning and dengue forecasting: Comparing random forests and artificial neural networks for predicting dengue burden at national and sub-national scales in Colombia | 2020 | Dengue | Colombia | 2014-2018 | Historical dengue data, environmental and meteorological variables, and socio-demographic variables. | Forecasting | E, H | Epi, Demo, Soc Eco, Clim, Geo | ANN, ARIMA, RF | RF | National and local (department-specific) models were compared and used to predict future dengue cases in Colombia. The results showed that RF more accurately estimated the counts of future dengue cases than ANN. Environmental and meteorological predictors were more significant for short-term forecast horizons, while sociodemographic predictors played a larger role in longer-term forecast horizons. |
| Zheng et al.  (151) | Seasonality modeling of the distribution of Aedes albopictus in China based on climatic and environmental suitability | 2019 | Mosquito-borne diseases | China | 1998-2017 | Ae. albopictus field surveillance data (1998-2017), meteorological data, climatic zones and spatial distribution | Risk mapping, Vector/host ecology | A, E | Clim, Ento | CART, k-medoids | CART | The study developed models predicting the seasonal distribution of Aedes albopictus in China, highlighting its potential range expansion based on climatic and environmental factors. The study found that Ae. albopictus could occur seasonally in semiarid or even arid areas, and its range expansion is linked to climate change. The predicted regions with suitable climates from May to September extended beyond the areas predicted in other recent studies. |

*Abbreviations were used for the One Health domain: E = Environment, A=Animal, H= Human; and for predictor types: Epi = Epidemiological, Clim = Climate, Demo= Demographic, Soc-Eco = Socio-economic, Geo = Geographic, Ento= Entomological, Net = Internet-based, O = Others ( the specific predictor is mentioned in this case).*

*Supp.2 Traffic light graph with the results of the risk of bias assessment.*
